# High Fat Diet Decreases Neuronal Activation in the Brain Induced by Resistin and Leptin

**DOI:** 10.3389/fphys.2017.00867

**Published:** 2017-11-28

**Authors:** Naif Alsuhaymi, Hamza Habeeballah, Martin J. Stebbing, Emilio Badoer

**Affiliations:** Neuropharmacology and Neuroinflammation, School of Health and Biomedical Sciences, RMIT University, Melbourne, VIC, Australia

**Keywords:** resistin, leptin, neuronal activation, high fat diet, central interactions

## Abstract

Resistin and leptin are adipokines which act in the brain to regulate metabolic and cardiovascular functions which in some instances are similar, suggesting activation of some common brain pathways. High-fat feeding can reduce the number of activated neurons observed following the central administration of leptin in animals, but the effects on resistin are unknown. The present work compared the distribution of neurons in the brain that are activated by centrally administered resistin, or leptin alone, and, in combination, in rats fed a high fat (HFD) compared to a normal chow diet (ND). Immunohistochemistry for the protein, Fos, was used as a marker of activated neurons. The key findings are (i) following resistin or leptin, either alone or combined, in rats fed the HFD, there were no significant increases in the number of activated neurons in the paraventricular and arcuate nuclei, and in the lateral hypothalamic area (LHA). This contrasted with observations in rats fed a normal chow diet; (ii) in the OVLT and MnPO of HFD rats there were significantly less activated neurons compared to ND following the combined administration of resistin and leptin; (iii) In the PAG, RVMM, and NTS of HFD rats there were significantly less activated neurons compared to ND following resistin. The results suggest that the sensitivity to resistin in the brain was reduced in rats fed a HFD. This has similarities with leptin but there were instances where there was reduced sensitivity to resistin with no significant effects following leptin. This suggests diet influences neuronal effects of resistin.

## Introduction

Resistin and leptin are adipokines synthesized in adipose tissue and both have actions in the brain to modulate metabolic functions, such as food intake and energy homeostasis, and they also influence cardiovascular regulation.

Resistin, first described in 2001 by Steppan et al. (2001) has been shown to act in the brain to reduce food intake and thermogenesis. However, it increases sympathetic nerve activity to the muscle vasculature (Kosari et al., 2011) and to the kidneys (Kosari et al., 2012). The effect on renal sympathetic nerve activity (RSNA) is mediated by phosphatidylinositol 3 kinase (PI 3-kinase) (Kosari et al., 2011, 2012).

Leptin is a much better characterized hormone than resistin. A primary role of leptin in healthy weight individuals is to reduce food intake by suppressing appetite (Friedman, 1998). Combined with its actions to increase energy expenditure and thermogenesis, leptin's effect is to reduce weight (Haynes et al., 1997a; Rahmouni et al., 2009).

Leptin can also influence cardiovascular function; intracerebroventricular (ICV) injection of leptin increases sympathetic nerve activity to the muscle and splanchnic vasculature (Haynes et al., 1997b) as well kidneys (Dunbar et al., 1997), a key organ for long term blood pressure control (Bełtowski, 2006). The mechanisms responsible for the increase in RSNA induced by leptin involve phosphatidylinositol 3 kinase (PI 3-kinase) (Rahmouni et al., 2003).

Thus, resistin and leptin have similar actions on cardiovascular outputs like RSNA but opposing actions on metabolic outputs like thermogenesis. This suggests some similarity in the brain sites of action of resistin and leptin, as well as differences.

A primary site of action of leptin is the hypothalamic arcuate nucleus (ARC) (Elias et al., 1998) but other brain nuclei are also affected by leptin as shown by studies using microinjections of leptin or the studies describing the distribution of its receptor (Van Dijk et al., 1996; Woods and Stock, 1996; Elias et al., 2000). In contrast, the sites of action for resistin in the brain have not been extensively studied. However, potential sites of action have been investigated using the detection of the protein, Fos, as a marker of activated neurons (Tovar et al., 2005; Singhal et al., 2007; Kosari et al., 2011; Habeeballah et al., 2016). The studies suggest that resistin and leptin may activate similar nuclei in the hypothalamus and medulla oblongata (Habeeballah et al., 2016).

In metabolic disorders induced by high fat diets, studies investigating the distribution of Fos following central leptin administration have shown a reduction in the number of activated neurons in several hypothalamic areas involved in metabolic and cardiovascular regulation such as the paraventricular nucleus (PVN), supraoptic nucleus (SON), arcuate nucleus (ARC), and the dorsomedial hypothalamus (DMH) (Prior et al., 2010), which may correlate with the reported attenuation of the anorexigenic actions of leptin (Rahmouni et al., 2005). Thus, high fat diets can influence the distribution of activated neurons following centrally administered leptin but the effect of diets high in fat on the distribution of Fos in response to resistin has not been investigated. This may be very important since the combined actions of resistin and leptin induce markedly greater effects on RSNA than either alone (Habeeballah et al., 2016), suggesting central interactions of these two adipokines. Whether this interaction is also influenced by dietary fat has not been explored.

The aims of the present work, therefore, were to determine the effect of high fat diets on the distribution in the brain of neurons activated by centrally administered (i) resistin (ii) leptin, and (iii) resistin combined with leptin.

## Methods

### Ethics statement

All the experimental protocols were performed in accordance with the Prevention of Cruelty to Animals Act 1986 (Australia). The procedures conform to the “Guiding Principles for Research Involving Animals and Human Beings” and the guidelines set out by the Australian Code of Practice for the Care and Use of Animals for Scientific Purposes, 2013 (National Health and Medical Research Council of Australia) and were approved by the RMIT University Animal Ethics Committee.

### Animal

Male Sprague-Dawley rats were obtained from the Animal Resources Centre (ARC; WA, Australia). The rats were caged in pairs at 23°C with a 12 h−12 h light-dark cycle at the Animal Facility located at RMIT University, Victoria, Australia. They were divided into two groups; one was fed a high-fat diet (HFD) (22% fat; Specialty Feeds, Glen Forrest, WA) and the other group was fed normal chow (normal diet, 4.8% fat) (ND). The body weights and food consumption were measured weekly for a period of 8 weeks.

### Fat percentage measurement

At the end of the feeding regime, whole-body fat composition was measured with magnetic resonance imaging (MRI) using an EchoMRI™ machine (EchoMRI LLC; Houston, Texas, US). Each rat was placed into a clear plastic tubular holder which was inserted into the EchoMRI™ machine. Scans averaged 1–2 min in duration. The fat content was determined and adjusted for body weight to yield the body fat percentage for each rat.

### Experimental protocol

#### Microinjections into the lateral brain ventricle

On the day of the experiment, anesthesia was induced using isoflurane gas (initially 5% then lowered to 2.5%) in O_2_ and maintained with intravenous urethane (1.4–1.6 g/kg initially, followed by supplemental doses of 0.05 ml of a 25% solution, as required) as described (Habeeballah et al., 2016). The depth of anesthesia was maintained to ensure the absence of corneal and pedal reflexes. Rats were kept on a heating pad for the duration of the experiments to maintain body temperature at ~37.5°C.

After shaving the head, the rat was positioned in a prone position, with the head placed in a Stoelting stereotaxic frame (Stoelting; Wood Dale, Illinois, US). The skull was exposed by a midline incision and bregma and lambda were positioned on the same horizontal plane. A small hole (2.0 mm) was drilled 1.4 mm lateral from the midline and 0.7 mm caudal to bregma to expose the surface of the brain. A fine glass micropipette (50–70 μm tip diameter) was inserted into the lateral brain ventricle (3.9 mm ventral to the surface of the dura) to inject the adipokines (resistin (7 μg in 5 μl of saline), leptin (7 μg in 5 μl of saline), resistin and leptin combined or saline (5 μl). The renal nerves were exposed and recorded and the data in rats fed the normal chow has been reported previously (Habeeballah et al., 2016).

#### Perfusion and tissue collection

Three hours after intracerebroventricular injection (ICV) the animals were overdosed with pentobarbitone (325 mg i.v.) and perfused transcardially with 200 ml of 0.1 M phosphate buffered saline (PBS) then 200 ml of 4% Paraformaldehyde (PFA). Then the brain was removed and post-fixed in 4% PFA for ~3 h and left overnight in 0.1 M PBS containing 20% sucrose. The white adipose tissue (epididymal fat) around the testicles was also removed and weighed.

#### Immunohistochemistry

Serial coronal sections (40 μm thick) were cut using a cryostat (Leica, CM1900, Germany). Immunohistochemistry was performed on free-floating sections. One in five sections was taken and placed in a 24-well plate. Briefly, immunohistochemistry was performed to detect the protein Fos, which is a marker of neuronal activation. The process involved incubation of the sections in 0.5% H_2_O_2_ (in 0.1 M PBS) for 15 min to destroy endogenous peroxidase activity followed by three 5 min washes with 0.1 M PBS. To enable antibody penetration, the sections were incubated with 0.5% Triton X and 10% normal horse serum (NHS) in 0.1 M PBS for 60 min. Following washes, the sections were incubated overnight with anti Fos primary antibody [rabbit polyclonal IgG, c-Fos (K-25): sc-253, Santa Cruz Biotechnology, CA, USA] dilution 1:5,000 in 0.1 M PBS containing 2% NHS and 0.3% Triton X. The following day, the sections were washed in 0.1 M PBS and incubated with biotinylated secondary antibody (polyclonal anti-rabbit, Vector Laboratories) raised in goat (1:400 in 0.1 M PBS containing 2% NHS) for 90 min. After washing with 0.1 M PBS, the sections were incubated with ExtrAvidin (Sigma Aldrich; St. Louis, Missouri, US) (1:400 in 0.1 M PBS containing 2% NHS) for 45 min. Following more washes the sections were incubated for 10 min in 0.1 M PBS containing 0.01% 3,3′-diaminobenzidine hydrochloride (Sigma life science), 0.02% NH_4_NiSO_3_ and 0.02% CoCl. Five microliters of 30% hydrogen peroxide was subsequently added and the reaction was terminated 15 min later by adding excess 0.1 M PBS. The sections were then mounted onto gelatine-coated glass slides. The sections were allowed to dry overnight at room temperature, then dehydrated in a series of ethanol solutions of increasing concentration (50–90% (w/v), followed by dipping into xylene. Coverslips were fixed onto the slides using DePex (Thermo fisher scientific, Melbourne, Australia) as a mounting medium.

#### Fos counting and photomicroscopy

The Fos-positive cell nuclei were counted in two sections of the organum vasculosum of the lamina terminalis (OVLT), median preoptic nucleus (MnPO), supraoptic nucleus (SON), arcuate nucleus (ARC), dorsomedial hypothalamic nuclus (DMH), and rostral ventromedial medulla (RVMM); in four sections of the paraventricular nucleus (PVN), lateral hypothalamic area (LHA), nucleus of the solitary tract (NTS), Raphe pallidus (RPA), and rostral ventrolateral medulla (RVLM); and in six sections of the periaquaductal gray (PAG), and Dorsal raphe (DR). Counting was performed unilaterally in each brain area except those located in the midline (RPA, DR, MnPO, and OVLT). All brain areas counted are shown schematically in supplementary figures (Supplementary Figures I–VIII, Supplementary File 1). Photographic images of each region (× 200 magnification) shown in the supplementary figures were obtained for counting purposes using a digital camera (Sensi Cam, PCO CCD Imaging, Kelheim, Germany) attached to an Olympus BX60 microscope (Olympus Inc.; Center Valley, Pennsylvania, US). The number of Fos-positive cell nuclei in rats fed a normal chow diet has been described previously for some of the brain areas (Habeeballah et al., 2016). For comparison of normal and high fat diets the data from that study using rats fed a normal diet, has been used in the present study for the OVLT, MnPO, ARC, and SON but with the addition of one more animal to the control group. In the PVN, DR, NTS, RPA, and RVLM, counting for the present analysis was performed in twice as many sections as in our previous work (Habeeballah et al., 2016). For the LHA, PAG, and RVMM of rats fed normal chow the data has not been reported previously. All data from rats fed the high fat diet has not been reported previously. It should be noted that the ND and HFD dietary treatments were run concurrently.

#### Statistical analysis

The average number of Fos-positive cell nuclei was compared between drug treatment groups within diet by using one-way ANOVA. Multiple comparisons were accounted for by the Holm-Sidak test. Between diets, the average number of Fos-positive cell nuclei were compared by using unpaired *t*-test and the *P*-value was adjusted for multiple comparisons using the Holm-Sidak test.

## Results

### Effects of resistin and leptin alone or combined on the distribution of fos-positive cell nuclei in rats fed a high fat diet (HFD)

#### OVLT and MnPO

**Figure 1:**
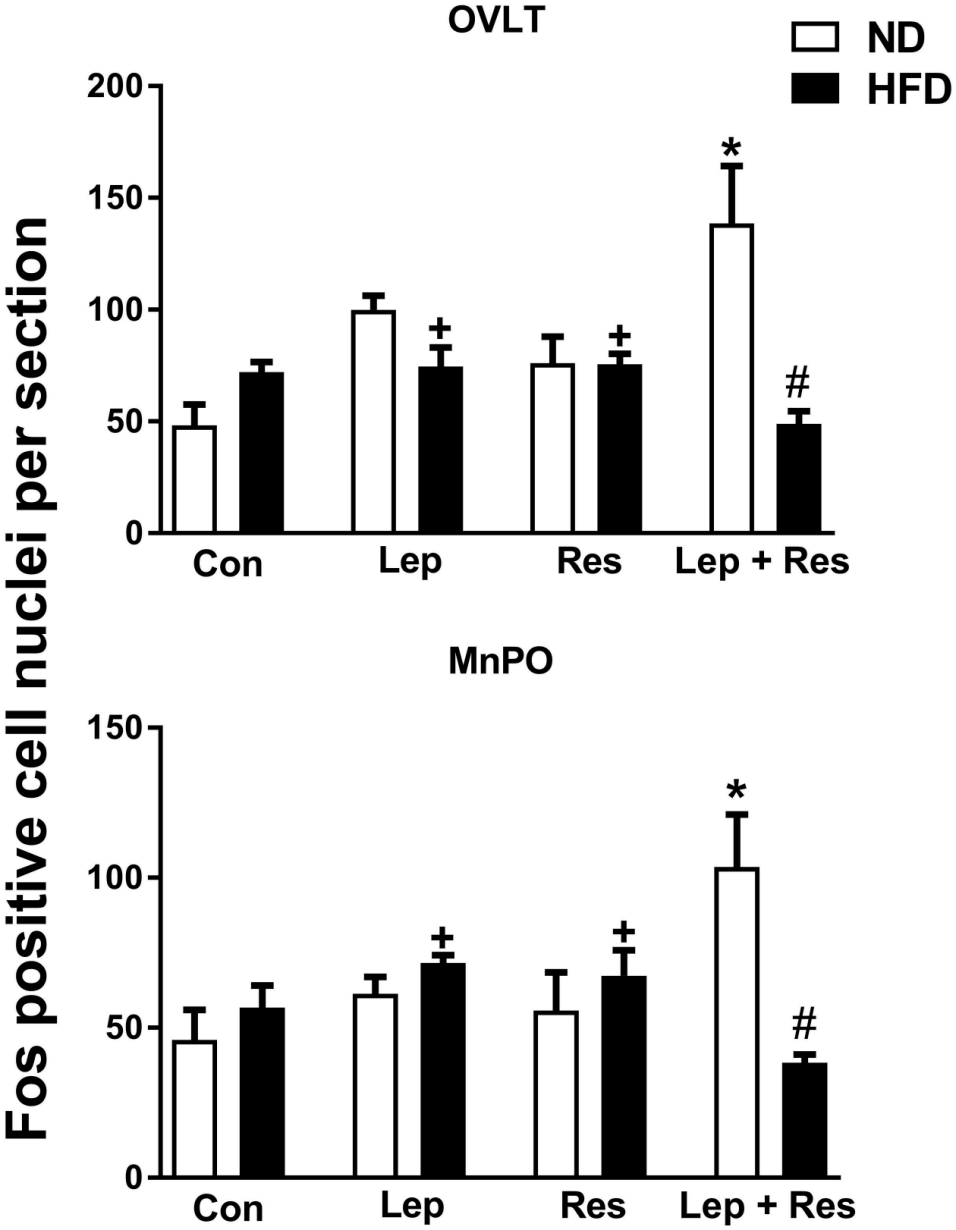
Numbers of Fos-positive cell nuclei per section in the organum vasculosum of the lamina terminalis (OVLT) and median preoptic nucleus (MnPO) from rats fed a normal chow (ND) and a high fat diet (HFD) administered intracerebroventricular saline (control, *n* = 6 ND, *n* = 4 HFD; 5 μl), leptin (*n* = 5 ND, *n* = 5 HFD; 7 μg in 5 μl), resistin (*n* = 4 ND, *n* = 4 HFD; 7 μg in 5 μl) or leptin combined with resistin (*n* = 5 ND, *n* = 6 HFD). *P < 0.005 and *P* < 0.05 compared with control in the OVLT and MnPO, respectively. ^#^*P* < 0.005 HFD compared to ND in the OVLT and MnPO. ^+^*P* < 0.05 and *P* < 0.005 compared with leptin + resistin in the OVLT and MnPO respectively.

When compared to controls, there was no significant effect of the hormones in the OVLT or MnPO in rats fed the HFD (Figure 1). However, the combination of resistin and leptin exhibited significantly lower numbers of Fos-positive cell nuclei compared to resistin or leptin administered alone in both the OVLT (*P* < 0.05) and MnPO (*P* < 0.005) (Figure 1).

#### Hypothalamus

**Figure 2:**
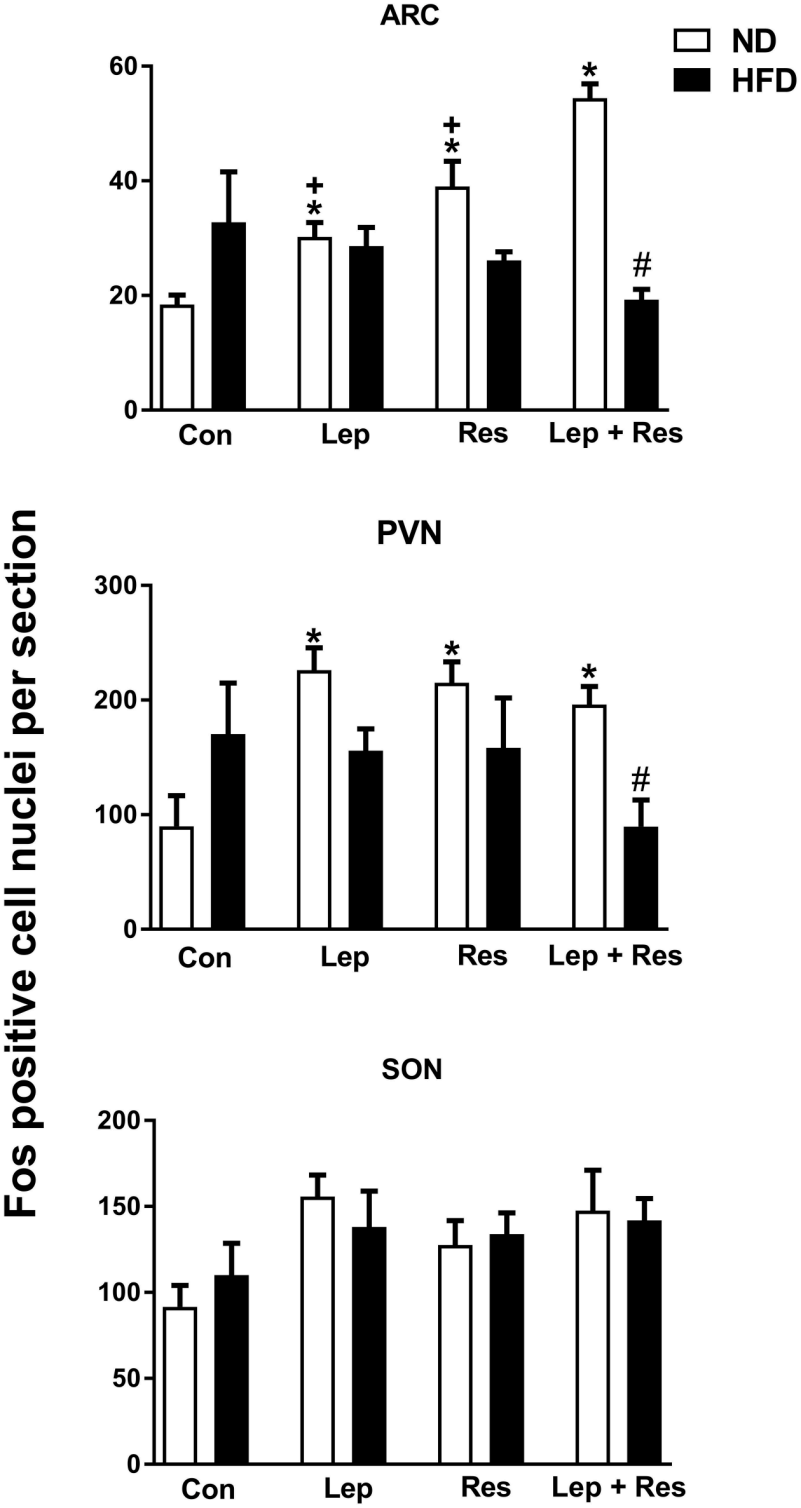
Numbers of Fos-positive cell nuclei counted unilaterally per section in the supraoptic nucleus (SON), arcuate nucleus (ARC) and paraventricular nucleus (PVN) from rats fed a normal chow (ND) and a high fat diet (HFD). Rats were administered intracerebroventricular saline (control, *n* = 6 ND, *n* = 4 HFD; 5 μl), leptin (*n* = 5 ND, *n* = 5 HFD; 7 μg in 5 μl), resistin (*n* = 4 ND, *n* = 4 HFD; 7 μg in 5 μl) or leptin combined with resistin (*n* = 5 ND, *n* = 6 HFD). In the paraventricular nucleus (PVN) for saline (*n* = 4 ND) and leptin (*n* = 4 ND) due to technical issues. *0.0001 < *p* < 0.005 compared with control in the ARC; *0.005 < *p* < 0.01 compared with control in the PVN; ^#^*P* < 0.0001 and ^#^*P* < 0.01 HFD compared to ND in the ARC and PVN respectively; ^+^0.0001 < *p* < 0.005 compared with leptin + resistin.

**Figure 3:**
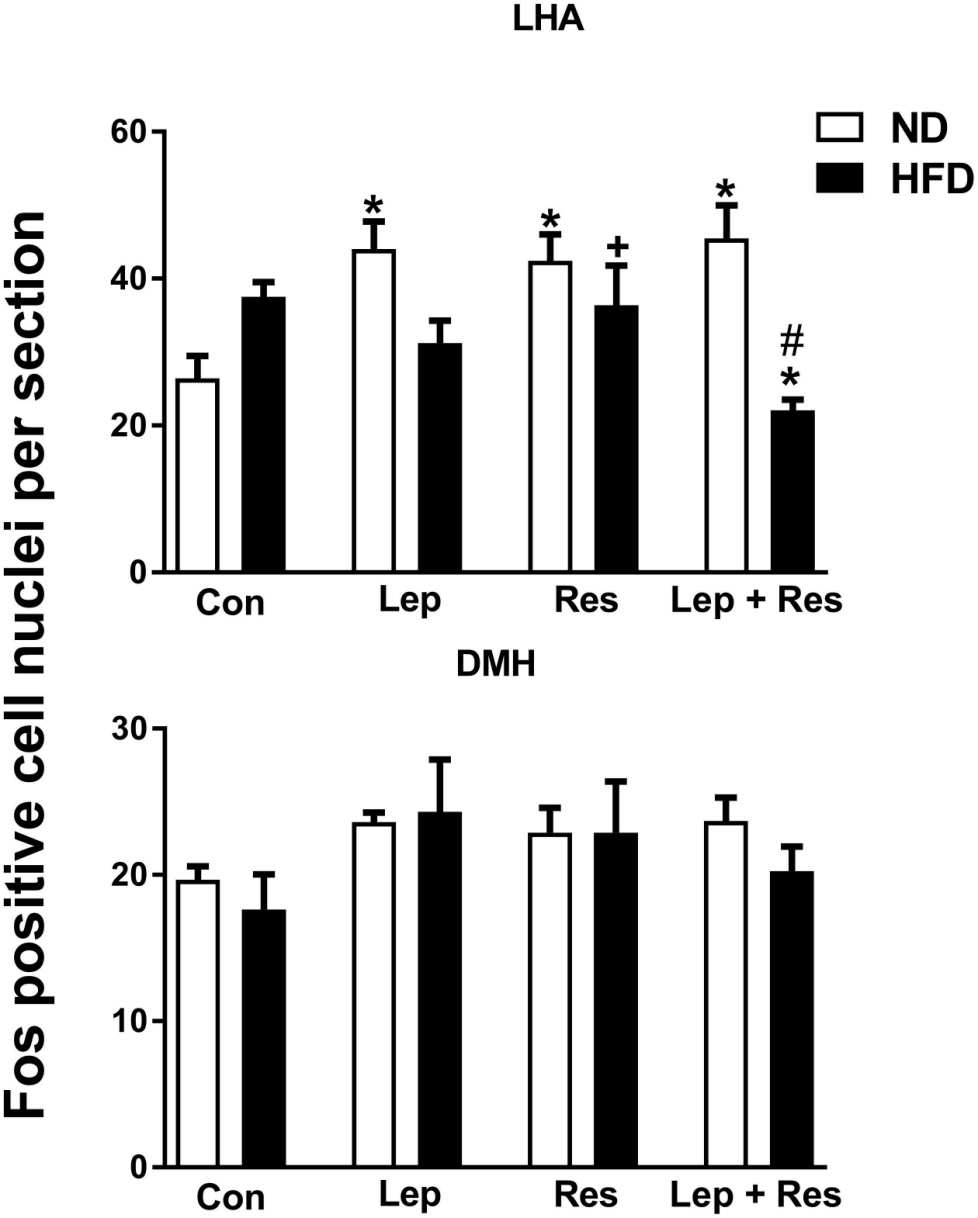
**Upper:** Numbers of Fos-positive cell nuclei counted unilaterally per section in the lateral hypothalamic area (LHA) from rats fed a normal chow (ND) and a high fat diet (HFD) administered intracerebroventricular (ICV) saline (control, *n* = 4 ND, *n* = 4 HFD; 5 μl), leptin (*n* = 4 ND, *n* = 5 HFD; 7 μg in 5 μl), resistin (*n* = 4 ND, *n* = 4 HFD; 7 μg in 5 μl), or leptin combined with resistin (*n* = 5 ND, *n* = 6 HFD). *P < 0.05 compared with control. ^#^*P* < 0.0005 HFD compared to ND. ^+^*P* < 0.05 hormone alone compared with leptin + resistin. **Lower:** Numbers of Fos-positive cell nuclei counted unilaterally per section in the the dorsomedial hypothalamic nuclei (DMH) administered ICV saline (control, *n* = 6 ND, *n* = 4 HFD; 5 μl), leptin (*n* = 5 ND, *n* = 5 HFD; 7 μg in 5 μl), resistin (*n* = 4 ND, *n* = 4 HFD; 7 μg in 5 μl) or leptin combined with resistin (*n* = 5 ND, *n* = 6 HFD).

In the high fat fed animals, there were no significant differences in the number of Fos-positive cell nuclei present in the ARC, PVN, SON, and DMH following resistin and leptin alone or combined (Figures 2, 3). In the LHA, although resistin or leptin alone did not significantly affect the number of Fos-positive cell nuclei compared to control (Figure 3), when both adipokines were combined there was a significant reduction in the number of Fos-positive cell nuclei compared to control (*P* < 0.05), and resistin alone (*P* < 0.05) (Figure 3).

#### Periaqueductal gray (PAG) and dorsal raphe (DR)

**Figure 4:**
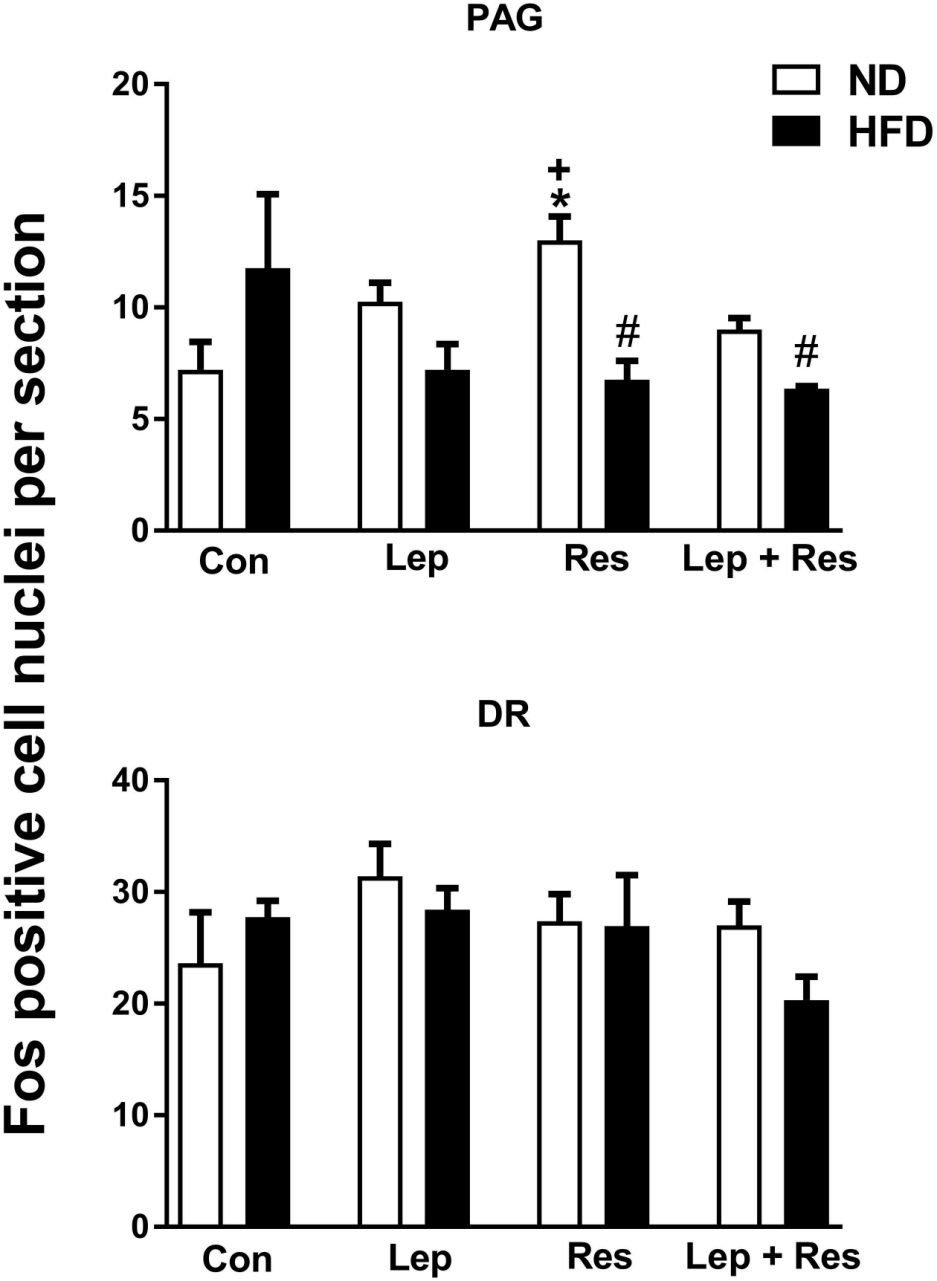
Numbers of Fos-positive cell nuclei counted unilaterally per section in the periaquaductal gray (PAG) and Dorsal raphe (DR) from rats fed a normal chow (ND) and a high fat diet (HFD) administered intracerebroventricular saline (control, *n* = 4 ND, *n* = 4 HFD; 5 μl), leptin (*n* = 4 ND, *n* = 5 HFD; 7 μg in 5 μl), resistin (*n* = 4 ND, *n* = 4 HFD; 7 μg in 5 μl), or leptin combined with resistin (*n* = 5 ND, *n* = 6 HFD). *P < 0.005 compared with control. ^#^*P* < 0.005 HFD compared to ND. ^+^*P* < 0.05 hormone alone compared with leptin + resistin.

In rats fed the HFD there were no significant differences in the number of Fos-positive cell nuclei in the PAG or the DR following resistin and leptin alone or combined (Figure 4).

#### Medulla oblongata

**Figure 5:**
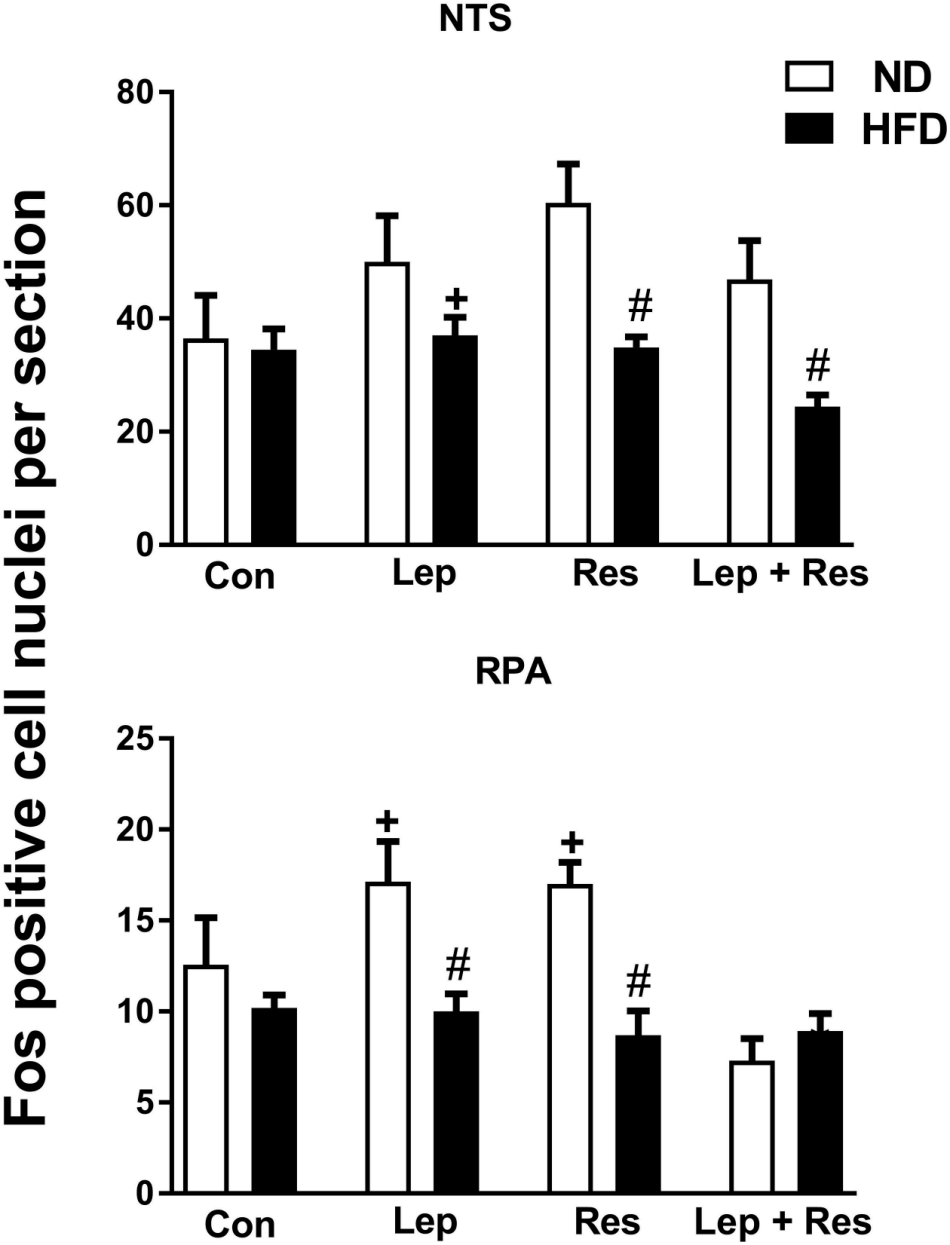
Numbers of Fos-positive cell nuclei counted unilaterally per section in the nucleus of the solitary tract (NTS) and Raphe pallidus nuclei (RPA) from rats fed a normal chow (ND) and a high fat diet (HFD) administered intracerebroventricular saline (control, *n* = 4 ND, *n* = 4 HFD; 5 μl), leptin (*n* = 4 ND, *n* = 5 HFD; 7 μg in 5 μl), resistin (*n* = 4 ND, *n* = 4 HFD; 7 μg in 5 μl), or leptin combined with resistin (*n* = 5 ND, *n* = 6 HFD). ^#^0.01 < *p* < 0.05 HFD compared to ND in the NTS; ^#^0.005 < *P* < 0.05 HFD compared to ND in the RPA; ^+^ *P* < 0.05 and *P* < 0.01 hormone alone compared with leptin + resistin for NTS and RPA respectively.

ICV injection of resistin or leptin alone or combined did not significantly affect the number of Fos-positive cell nuclei in the NTS compared to control in rats fed the HFD (Figure 5). The combination of resistin and leptin, however, resulted in a significantly lower number compared to leptin alone (*P* < 0.005) but not compared to resistin alone (Figure 5).

**Figure 6:**
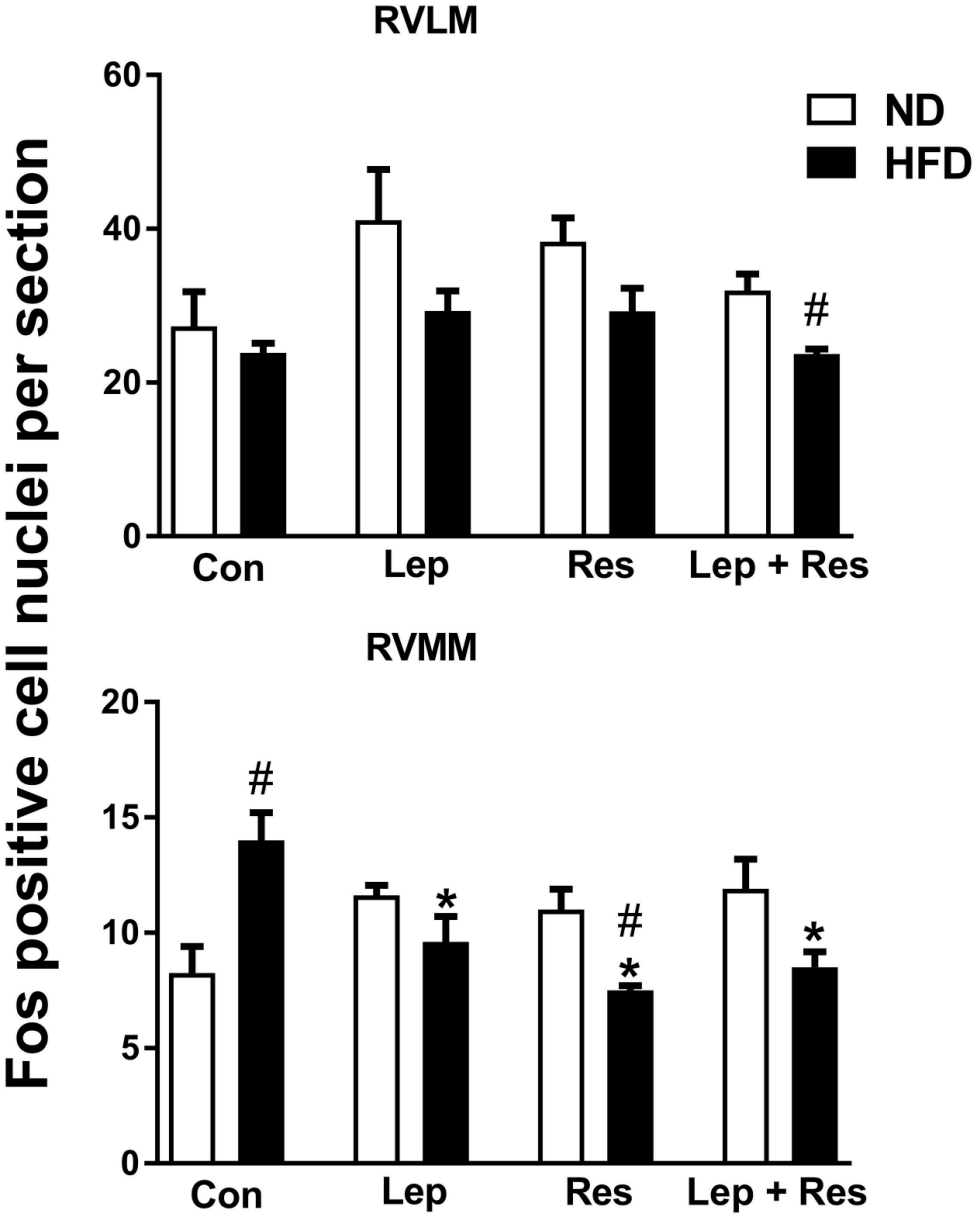
Numbers of Fos-positive cell nuclei counted unilaterally per section in the rostral ventrolateral medulla (RVLM) and rostral ventromedial medulla (RVMM) from rats fed a normal chow (ND) and a high fat diet (HFD) administered intracerebroventricular saline (control, *n* = 4 ND, *n* = 4 HFD; 5 μl), leptin (*n* = 4 ND, *n* = 5 HFD; 7 μg in 5 μl), resistin (*n* = 4 ND, *n* = 4 HFD; 7 μg in 5 μl), or leptin combined with resistin (*n* = 5 ND, *n* = 6 HFD). *0.005 < *P* < 0.05 compared with control. ^#^0.005 < *P* < 0.05 HFD compared to ND.

In the RVMM there was a significant decrease in the number of Fos-positive cell nuclei following resistin (*P* < 0.005) and leptin (*P* < 0.05) alone or in combination (*P* < 0.005) compared to the controls in rats fed the HFD (Figure 6). The effects following the combination of resistin and leptin were not significantly different from either hormone alone (Figure 6).

In the both the RPA and RVLM there were no significant differences between the groups in rats fed HFD (Figures 5, 6).

### Comparison of the effects of resistin and leptin on fos-positive cell nuclei in rats fed the high fat diet vs. rats fed a normal diet

#### OVLT and MnPO

**Figure 7:**
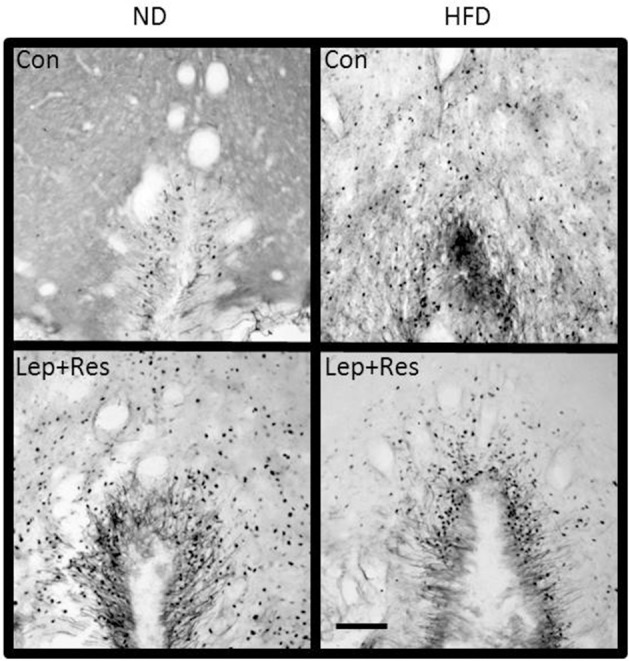
Photomicrographs showing Fos-positive cell nuclei in the organum vasculosum of the lamina terminalis of rats fed a high fat diet (HFD) and rats fed a normal diet (ND) following centrally administered saline (5 μl; control) or leptin combined with resistin (7 μg). Scale bar = 100μm. The rostrocaudal levels of the sections counted were at ~0.36–0.56 mm rostral of bregma.

**Figure 8:**
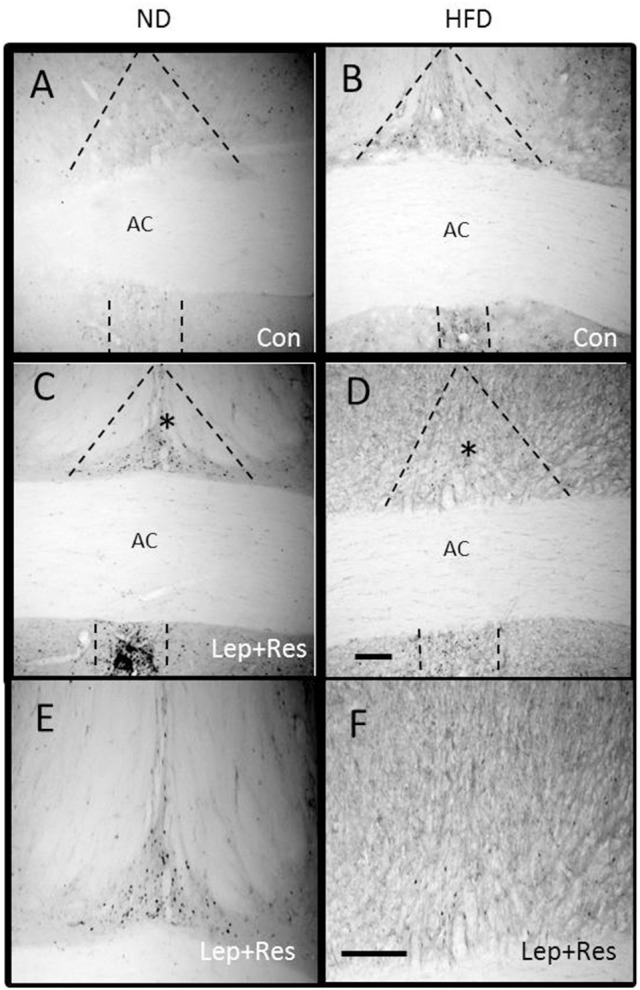
Photomicrographs showing Fos-positive cell nuclei in the median preoptic nucleus (outlined by dashed lines) of rats fed a high fat diet (HFD) and rats fed a normal diet (ND) following centrally administered saline (5 μl; control) or leptin combined with resistin (7 μg) **(A–D)**. ^*^Area is shown in higher magnification in **(E,F)** respectively. Scale bar = 100 μm. The rostrocaudal levels of the sections counted were at ~0.12–0.32 mm caudal of bregma.

The number of Fos positive-cell nuclei following resistin and leptin combined were significantly less compared to rats fed the ND in the OVLT (*P* < 0.005) (Figures 1, 7) and MnPO (*P* < 0.005) (Figures 1, 8). There were no significant differences between the diets in the number of Fos-positive cell nuclei present in the OVLT and MnPO when resistin or leptin were administered alone.

#### Hypothalamus

**Figure 9:**
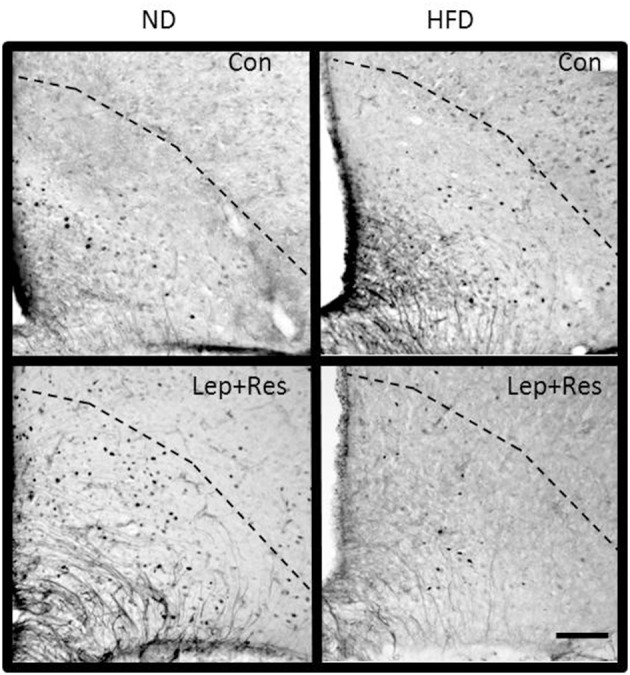
Photomicrographs showing Fos-positive cell nuclei in the arcuate nucleus (outlined by dashed lines) of rats fed a high fat diet (HFD) and rats fed a normal diet (ND) following centrally administered saline (5 μl; control) or leptin combined with resistin (7 μg). Scale bar = 100 μm. The rostrocaudal levels of the sections counted were at ~1.8–2.0 mm caudal of bregma.

**Figure 10:**
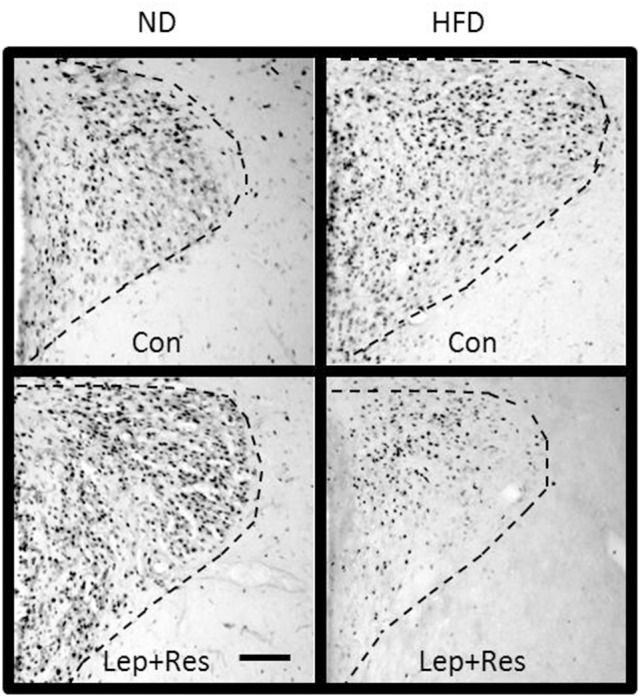
Photomicrographs showing Fos-positive cell nuclei in the paraventricular nucleus (outlined by dashed lines) of rats fed a high fat diet (HFD) and rats fed a normal diet (ND) following centrally administered saline (5 μl; control) or leptin combined with resistin (7 μg). Scale bar = 100 μm. The rostrocaudal levels of the sections counted were at ~1.32–1.52 mm caudal of bregma.

**Figure 11:**
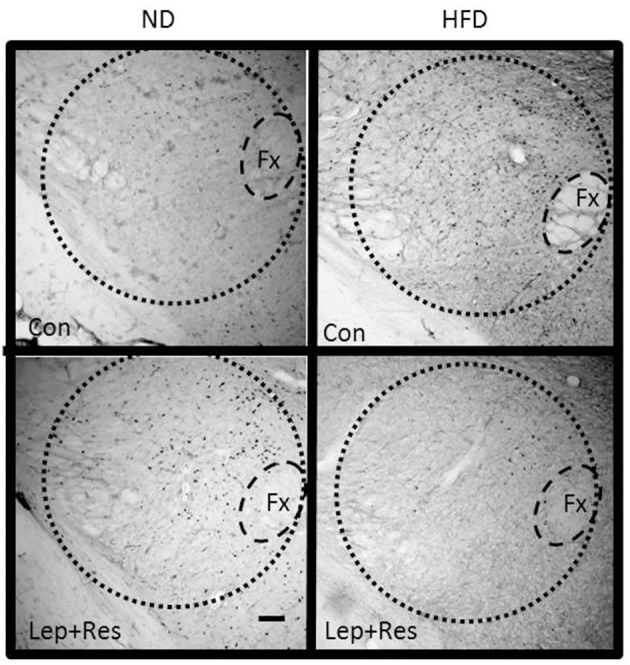
Photomicrographs showing Fos-positive cell nuclei in the lateral hypothalamic area (outlined by dashed lines) of rats fed a high fat diet (HFD) and rats fed a normal diet (ND) following centrally administered saline (5 μl; control) or leptin combined with resistin (7 μg). Fx, Fornix. Scale bar = 100μm. The rostrocaudal levels of the sections counted were at ~1.8–2.0 mm caudal of bregma.

The number of Fos-positive cell nuclei was significantly greater in rats fed a ND than in the HFD (*P* < 0.0001, *P* < 0.01, and *P* < 0.005 ARC, PVN, and LHA, respectively) (Figures 2, 3, 9–11). In contrast, there were no significant differences between the following each hormone alone. In the DMH and SON, there were no significant differences between diets (Figures 2, 3).

#### Periaqueductal gray (PAG) and dorsal raphe (DR)

**Figure 12:**
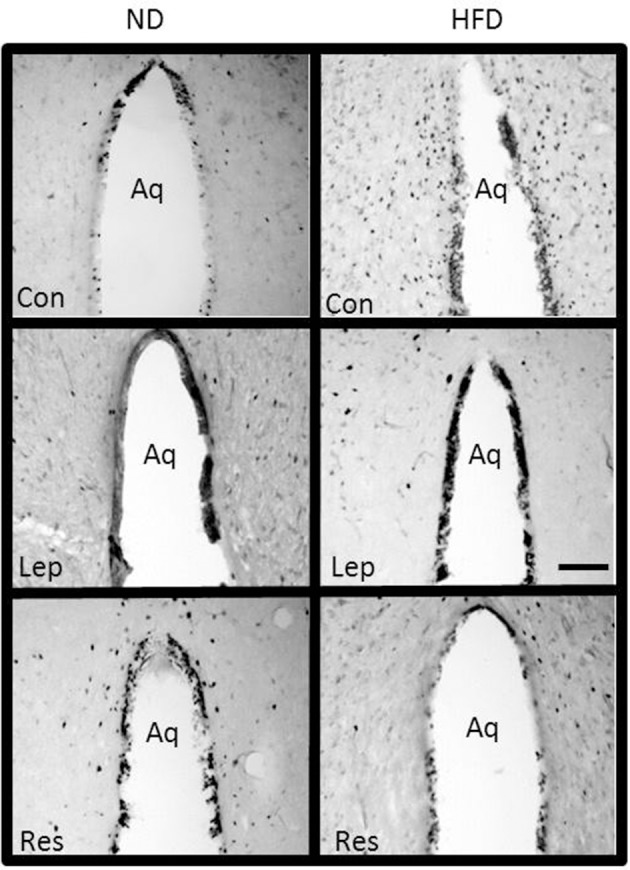
Photomicrographs showing Fos-positive cell nuclei in the periaquaductal gray surrounding the aqueduct (Aq) of rats fed a high fat diet (HFD) and rats fed a normal diet (ND) following centrally administered saline (5 μl; control), or leptin (7 μg) or resistin (7 μg). Scale bar = 100 μm. The rostrocaudal levels of the sections counted were at ~6.4–7.4 mm caudal of bregma.

The numbers of Fos-positive cell nuclei in the PAG following resistin alone and in combination with leptin were significantly lower in rats fed the HFD compared to ND (*P* < 0.005 and *P* < 0.005, respectively) (Figures 4, 12). There was no significant difference between the diets following leptin alone. In the DR, there were no significant differences in responses between the diets (Figure 4).

#### Medulla oblongata

**Figure 13:**
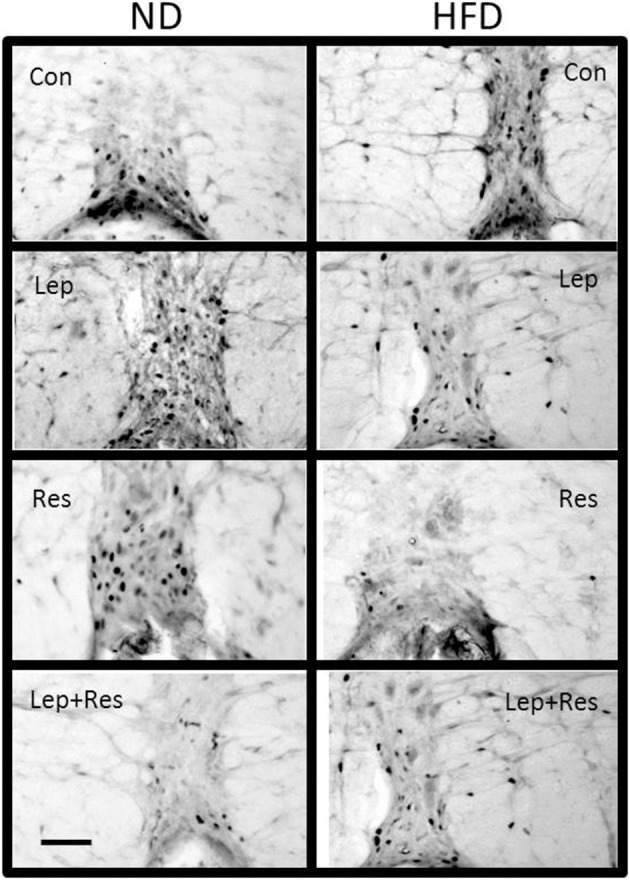
Photomicrographs showing Fos-positive cell nuclei in the Raphe pallidus of rats fed a high fat diet (HFD) and rats fed a normal diet (ND) following centrally administered saline (5 μl; control), leptin (7 μg), resistin (7 μg) or leptin combined with resistin. Scale bar = 100 μm. The rostrocaudal levels of the sections counted were at ~12.2–12.4 mm caudal of bregma.

In the RPA the numbers of Fos-positive cell nuclei following resistin (*P* < 0.001) or leptin alone (*P* < 0.05) were significantly less in rats fed the HFD compared to the ND (Figures 5, 13). When the hormones were combined there was no difference between the diets (Figure 5).

**Figure 14:**
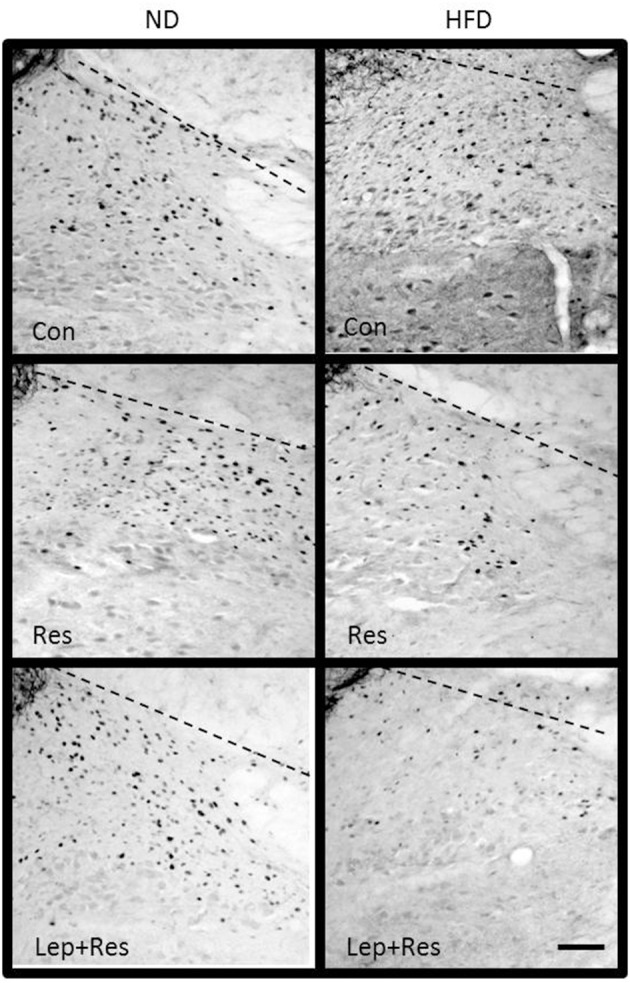
Photomicrographs showing Fos-positive cell nuclei in the nucleus of the solitary tract of rats fed a high fat diet (HFD) and rats fed a normal diet (ND) following centrally administered saline (5 μl; control), resistin (7 μg) or leptin combined with resistin. Scale bar = 100 μm. The rostrocaudal levels of the sections counted were at ~13.7–14.1 mm caudal of bregma.

In the NTS the effects of resistin alone (*P* < 0.01), and combined with leptin (*P* < 0.01), were significantly less in rats fed the HFD compared to the ND (Figures 5, 14). There was no difference following leptin alone between diets (Figure 5).

**Figure 15:**
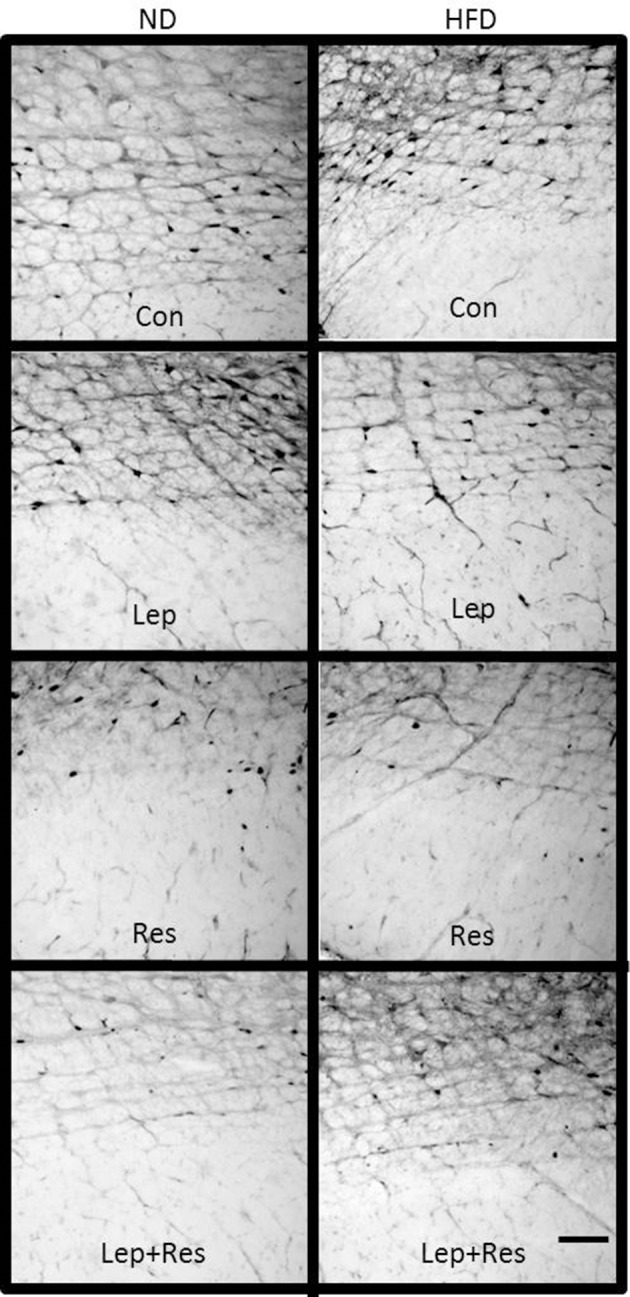
Photomicrographs showing Fos-positive cell nuclei in the rostral ventromedial medulla of rats fed a high fat diet (HFD) and rats fed a normal diet (ND) following centrally administered saline (5 μl; control) leptin (7 μg), resistin (7 μg) or leptin combined with resistin. Scale bar = 100 μm. The rostrocaudal levels of the sections counted were at ~12.2–12.4 mm caudal of bregma.

In the RVMM, only the effect of resistin alone was significantly less in rats fed the HFD compared to the ND (*P* < 0.01) (Figures 6, 15). Interestingly, in control rats fed the HFD, the number of Fos-positive cell nuclei was significantly greater than in the rats fed the ND (Figures 6, 15).

**Figure 16:**
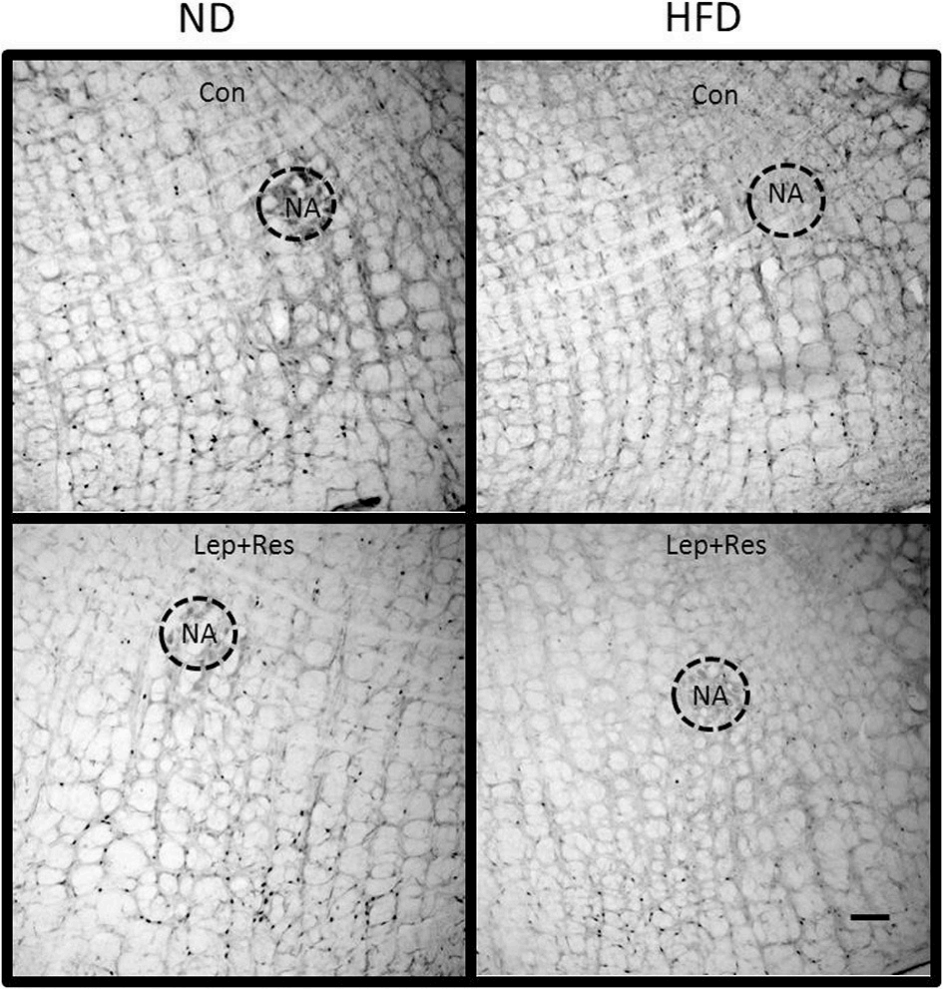
Photomicrographs showing Fos-positive cell nuclei in the rostral ventrolateral medulla of rats fed a high fat diet (HFD) and rats fed a normal diet (ND) following centrally administered saline (5 μl; control) or leptin combined with resistin (7 μg). NA, nucleus ambiguus. Scale bar = 100 μm. The rostrocaudal levels of the sections counted were at ~12.2–12.4 mm caudal of bregma.

In the RVLM only the response to resistin and leptin combined was significantly less in the HFD compared to the ND group (*P* < 0.005) (Figures 6, 16).

#### Metabolic parameters in rats fed HFD vs. ND

**Figure 17:**
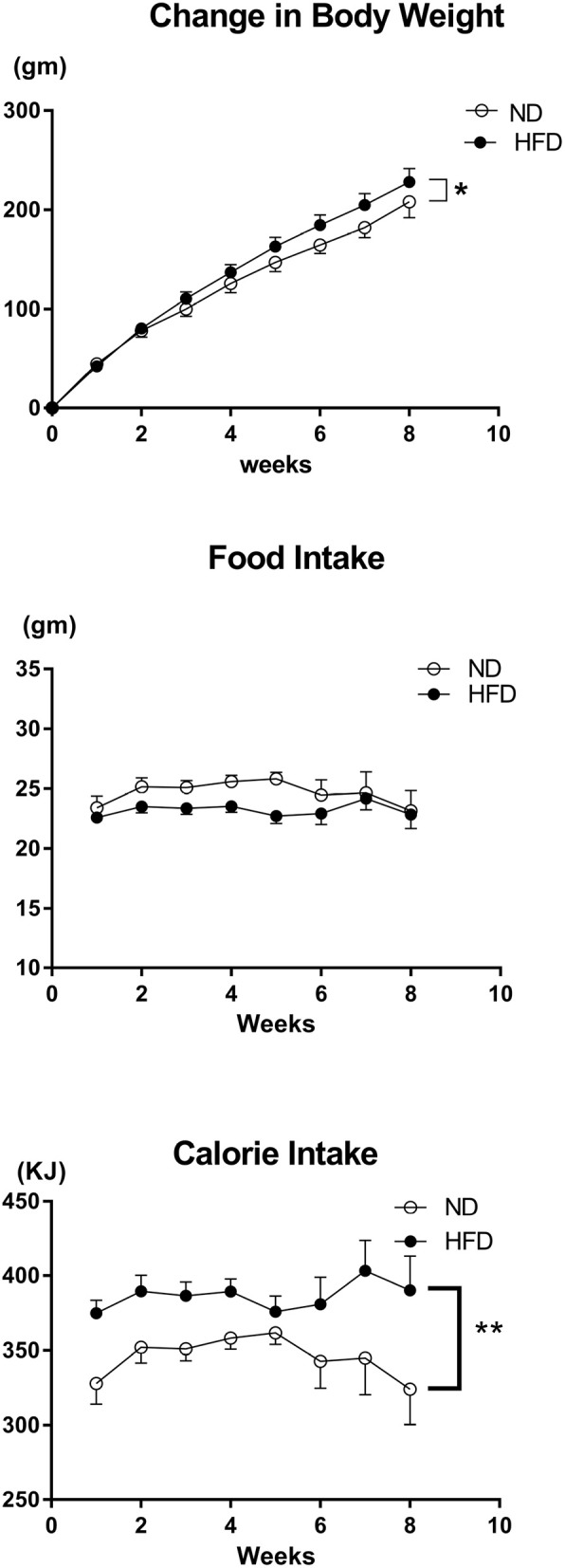
Effect of high fat diet (HFD; *n* = 18) and normal diet (ND; *n* = 13) on the change in body weight, food intake per day and average calorie intake per day over time. ^*^P < 0.01 compared with control. ^**^P < 0.0001 compared with control.

**Figure 18:**
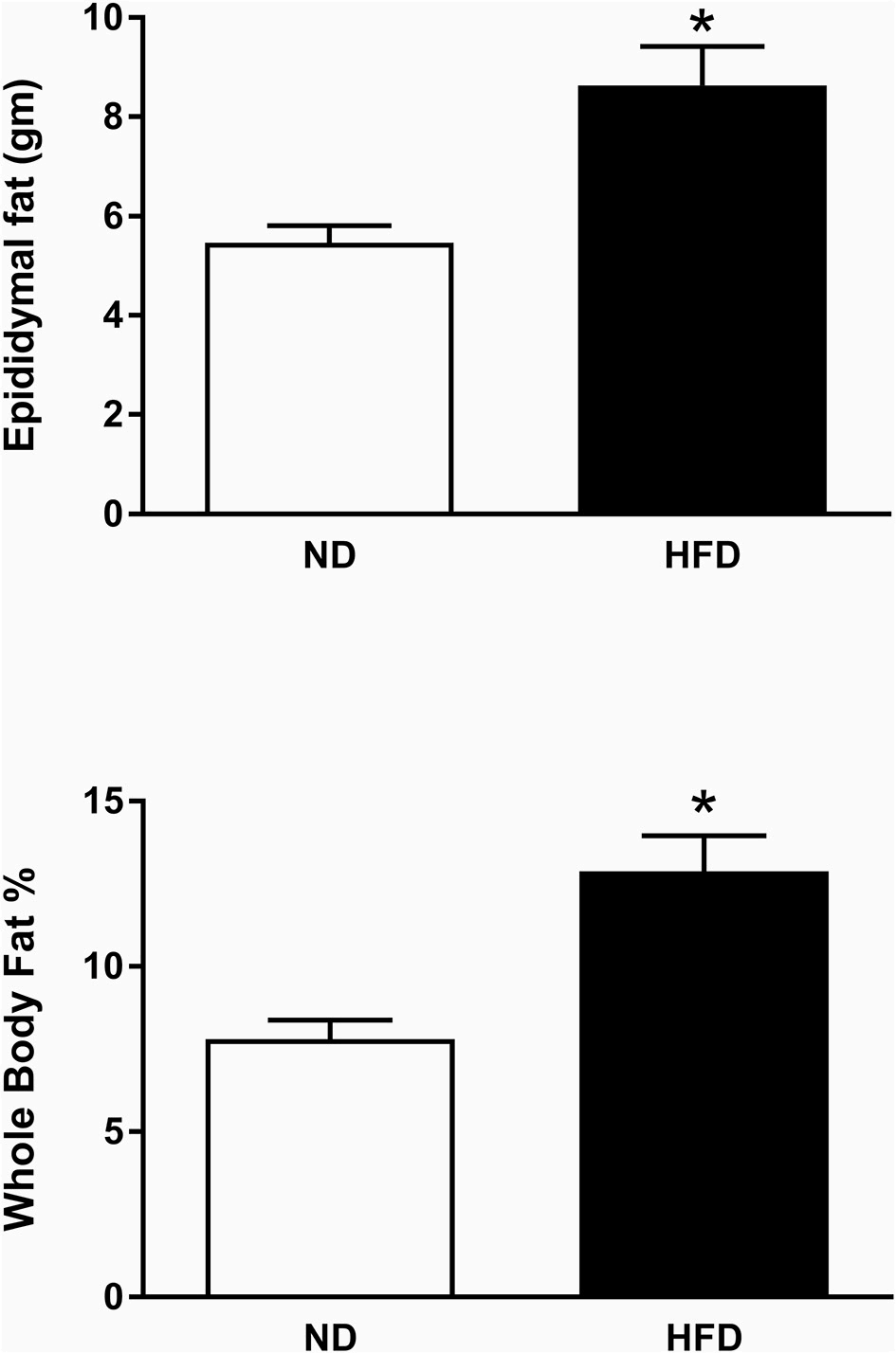
Effect of high fat diet (HFD) and normal diet (ND) in the epididymal fat (ND, *n* = 15 HFD, *n* = 15) and in the whole body fat percentage (ND, *n* = 10; HFD, *n* = 12). ^*^P < 0.001 compared with control.

The body weight prior to the start of the diet was on average 278 ± 5 g in ND group and 293 ± 6 g in the HFD group and there was no significant difference between the two groups. There was a significantly greater increase in body weight (*P* < 0.01) and calorie intake (*P* < 0.0001) in the HFD group compared to the ND group over time (Figure 17). However, there was no significant difference in food intake over time between the HFD and ND groups (Figure 17). Additionally, the percentage of whole-body fat (*P* < 0.001) and the amount of the epididymal fat (*P* < 0.001) at the end of the diet was significantly higher in rats fed the HFD compared with ND rats (Figure 18).

## Discussion

In the present work, we investigated the production of Fos as a marker of activated neurons in the brain following resistin and leptin alone or combined in rats fed a high fat diet and compared it to the effects in rats fed a normal diet. The findings are summarized in Table 1. The data show that high fat feeding reduced the number of activated neurons especially when resistin and leptin were combined in many brain nuclei examined. This suggests that the sensitivity to the effects of resistin and leptin were reduced.

**Table 1:** Summary of brain regions in which there were changes in the number of Fos-positive cell nuclei compared to control.

**Brain areas**	**Resistin**		**Leptin**		**Resistin** + **Leptin**	
	**ND**	**HFD**	**ND**	**HFD**	**ND**	**HFD**
OVLT	NSD	NSD	NSD	NSD	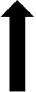	NSD
MnPO	NSD	NSD	NSD	NSD	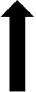	NSD
PVN	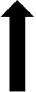	NSD	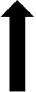	NSD	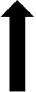	NSD
ARC	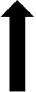	NSD	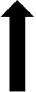	NSD	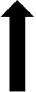	NSD
LHA	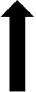	NSD	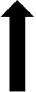	NSD	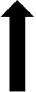	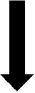
PAG	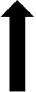	NSD	NSD	NSD	NSD	NSD
RVMM	NSD	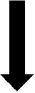	NSD	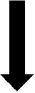	NSD	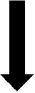

*NSD, No significant differences*.

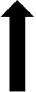
*Significant increase*.

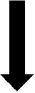
*Significant decrease*.

### HFD influences the neuronal activation induced by resistin and leptin alone

In rats fed a HFD, central resistin alone did not show any significant differences in the number of Fos-positive cell nuclei compared to the control in all brain regions that were examined, except in the RVMM in which central resistin showed a significant reduction. In contrast, rats fed a ND receiving central resistin alone showed a significant increase in Fos-positive cell nuclei in the PVN, ARC, LHA, and PAG. The findings confirm previous findings in which the PVN and ARC were examined (Singhal et al., 2007; Kosari et al., 2011). Thus, the present findings suggest that HFD can decrease the sensitivity to resistin in the brain.

Following the central administration of leptin there was a significant increase in the number of activated neurons in the PVN, ARC, and LHA in rats fed the ND as previously reported (Mercer et al., 1996; Van Dijk et al., 1996; Woods and Stock, 1996; Elias et al., 2000; Montanaro et al., 2005; Habeeballah et al., 2016). However, in rats fed a HFD, we found no significant differences from control in those areas, implying that the sensitivity of leptin was decreased by HFD feeding. Such a conclusion was also reached in a study using rabbits fed a high fat diet (Prior et al., 2010). The results in the ARC are particularly interesting given that the ARC is a key brain nucleus mediating the dietary effects of leptin. Thus, the decrease in Fos-positive cell nuclei in the ARC following central leptin administration corresponds with the decreased sensitivity to the anorexigenic actions of leptin in rats fed a HFD (Münzberg et al., 2004). It is unknown if similar functional changes elicited by central resistin, are affected by HFD.

In the RVMM of rats fed a HFD, we found that central resistin decreased the number of activated neurons in this brain region compared to control, although no differences were observed in the other areas of the medulla oblongata (i.e., RVLM, NTS, and RPA). Similarly, we found that central administration of leptin also decreased the number of activated neurons in the RVMM of rats fed a HFD.

A comparison between the HFD and ND shows significantly less activated neurons in the RVMM in the HFD group following resistin, suggesting that the sensitivity to resistin is reduced in this region as well. This interpretation is complicated, however, by the finding that in the control groups, there was a significantly greater number of Fos-positive cell nuclei in the RVMM in the HFD compared to the ND groups. Following leptin there were less activated neurons in the RVMM of the HFD compared to the ND group but this did not attain statistical significance.

Central resistin alone had no effect on Fos production in the PAG of rats fed a HFD compared to control but was able to increase it in rats fed a ND. Thus, in rats fed the HFD, the number of activated neurons was significantly less than in the ND group following resistin alone. In contrast, central leptin alone showed no changes in Fos production in both HFD and ND when compared to their respective controls. Thus, the PAG appears to be selectively activated following resistin administration to rats fed a ND, but this sensitivity was attenuated in the rats fed the HFD.

Similarly, when the effects in HFD and ND groups were compared, resistin or leptin alone in the HFD group elicited significantly less Fos production in the RPA. This nucleus plays a key role in thermogenesis by mediating sympatho-excitation to brown adipose tissue, thereby regulating non-shivering thermogenesis (Nakamura et al., 2004). However, since resistin and leptin have opposing actions on this output (Rahmouni et al., 2009; Kosari et al., 2011), the functional consequences of an attenuated sensitivity in the RPA to both hormones on thermogenesis needs to be investigated.

### HFD influences the neuronal activation induced by the combined treatment of resistin and leptin

In the HFD group resistin and leptin combined had no significant effect on the number of activated neurons in most areas examined (exceptions were in the LHA and RVMM) when compared to the saline control. This was in contrast to the findings in ND where the number of activated neurons increased in five brain regions (i.e., OVLT, MnPO, PVN, ARC, and LHA). Thus, in the HFD group, the numbers of activated neurons were significantly less than in the ND in each of those five brain nuclei. This data suggests that the sensitivity to the combined effects of resistin and leptin was decreased in these areas by the HFD.

Further, in specific brain regions the combination of resistin and leptin in the HFD group resulted in less activated neurons than either hormone alone (OVLT, MnPO), or resistin alone (LHA) or leptin alone (NTS). This suggests that in these brain regions there may be a complex interaction whereby the two hormones attenuate each other's actions.

### What are the mechanisms responsible for the sensitivity changes?

The mechanisms responsible for the decreased sensitivity to resistin in HFD are not known. For leptin, however, it has been reported that suppressor of cytokine signaling 3 (SOCS3) protein and protein tyrosine phosphatase 1B (PTP1B) negatively regulate leptin receptor signaling (Cheng et al., 2002; Zabolotny et al., 2002; Howard et al., 2004; Mori et al., 2004). In overweight/obese conditions, increased intracellular levels of SOCS3 and PTP1B appear to mediate the decreased sensitivity to the anorexigenic effects of leptin (Cheng et al., 2002; Zabolotny et al., 2002; Howard et al., 2004; Mori et al., 2004). It will be of interest to determine if similar intracellular mechanisms mediate the reduced sensitivity to resistin.

### Metabolic effects of the HFD

The metabolic measurements monitored in the present study show that there was a small but significantly greater weight gain in the rats fed the HFD compared to the ND. There was marked redistribution of adipose tissue with a 50% increase in percent body fat and a large increase in the amount of epididymal fat, even though the rats on the HFD ate less food. Our results suggest that the changes observed in the brain pathways activated by resistin and leptin can be observed without overt obesity.

### Limitations of the current study

In the present study, we have investigated the distribution of Fos-positive cell nuclei as a marker of activated neurons but it does not provide us with any information on whether the neurons are directly activated by the respective hormones or on the potential functional responses involved.

With respect to brain sites directly activated by resistin, there is little information since the receptors for resistin in the brain have not been identified. Thus, the findings are indicative of pathways that are activated following centrally administered resistin. These pathways may be related to the metabolic or the autonomic actions of resistin such as changes in RSNA (Kosari et al., 2011). In contrast, the receptors for leptin and their distribution in the brain have been well-documented (Mercer et al., 1996; Schwartz et al., 1996; Shioda et al., 1998).

Interpreting our findings with respect to potential functional roles is, of course, difficult. In the case of leptin, apart from the reduced anorexigenic effects, the responses to centrally administered leptin on sympathetic nerve activity to the lumbar region and to brown adipose tissue are also attenuated by HFD (Morgan et al., 2008). Thus, functional correlates exist and the reduced sensitivity shown by the decreased expression of Fos in the HFD are in agreement with the functional data known about leptin. However, for RSNA responses, there is no evidence of correlation. In particular, a greater increase in RSNA occurred following the combination of resistin and leptin than either hormone alone (Habeeballah et al., 2017). Thus, the current findings of reduced sensitivity following combined actions of resistin and leptin in HFD do not appear to be related to the RSNA responses.

Finally, anesthesia can influence the activity of neurons and this may limit our ability to determine the number of activated neurons. This could account for our inability to show a significant increase in the DMH as has been reported previously (Elmquist et al., 1997). However, this would suggest that brain regions in which we did observe significant changes may well be very important. Further, we have assumed that the effect of anesthesia is not influenced by diet.

## Conclusion

In conclusion, diets that are high in fat may lead to reduced sensitivity in the brain to resistin whether “alone” or when it is present with leptin. This is similar to the reduced sensitivity that has been observed previously with leptin in rats fed a HFD (Prior et al., 2010). The present study is in agreement with those reports. The reduced sensitivity to leptin correlates with the anorexigenic and sympathetic nerve responses to some tissues. Whether there are such functional correlates for resistin needs investigation.

## Author contributions

EB designed the study, analyzed and reviewed the data, designed the figures, and wrote the paper. NA performed the experiments, analyzed and reviewed the data, produced the figures, and wrote the paper. HH contributed to the experiments, reviewed the data and drafts of the paper. MS reviewed the data and contributed to drafts of the paper.

### Conflict of interest statement

The authors declare that the research was conducted in the absence of any commercial or financial relationships that could be construed as a potential conflict of interest.

## Supplementary material

Supplementary Figure I A schematic showing the approximate region of the organum vasculosum of the laminae terminalis (OVLT) in which Fos-positive cell nuclei were counted. The photomicrograph is an example of a section counted. The approximate rostrocaudal level relative to bregma is given in mm.

Supplementary Figure II A schematic showing the approximate region of the median preoptic nucleus (MnPO) in which Fos-positive cell nuclei were counted. The photomicrograph is an example of a section counted. The approximate rostrocaudal level relative to bregma is given in mm. ACA, anterior commissure.

Supplementary Figure III A schematic showing the approximate region of the supraoptic nucleus (SON) in which fos-positive cell nuclei were counted. The photomicrograph is an example of a section counted. The approximate rostrocaudal level relative to bregma is given in mm. Opt, optic tract.

Supplementary Figure IV A schematic showing the approximate region of the paraventricular nucleus (PVN) in which Fos-positive cell nuclei were counted. The photomicrograph is an example of a section counted. The approximate rostrocaudal level relative to bregma is given in mm. 3v, 3rd ventricle.

Supplementary Figure V A schematic showing the approximate region of the arcuate nucleus (ARC) in which fos-positive cell nuclei were counted. The photomicrograph is an example of a section counted. The approximate rostrocaudal level relative to bregma is given in mm.

Supplementary Figure VI A schematic showing the approximate region of the LHA and dorsomedial hypothalamus (DMH) in which fos-positive cell nuclei were counted. The photomicrograph is an example of a section counted. The approximate rostrocaudal level relative to bregma is given in mm.

Supplementary Figure VII A schematic showing the approximate region of the periaqueductal gray (PAG) and dorsal raphe nucleus (DR) in which fos-positive cell nuclei were counted. The photomicrograph is an example of a section counted. The approximate rostrocaudal level relative to bregma is given in mm.

Supplementary Figure VIII A schematic showing the approximate region of the RVLM, the medullary midline area Raphe pallidus (RPa) and rostral ventromedial medulla (RVMM) in which fos-positive cell nuclei were counted. The photomicrograph is an example of a section counted. The approximate rostrocaudal level relative to bregma is given in mm. AP, area postrema; ION, inferior olivary nucleus; NA, nucleus ambiguous; Pyr, pyramidal tract. All supplementary figures are modified from Paxinos and Watson (2004).

## Abbreviations

OVLT: organum vasculosum of the lamina terminalis
MnPO: median preoptic nucleus
PVN: paraventricular nucleus
ARC: arcuate nucleus
LHA: lateral hypothalamic area
PAG: periaquaductal gray
RVMM: rostral ventromedial medulla.
