# Supplementary material for: High Fat Diet Decreases Neuronal Activation in the Brain Induced by Resistin and Leptin
Source: Front Physiol. 2017 Nov 28;8:867. doi: 10.3389/fphys.2017.00867 (PMC5712409; doi:10.3389/fphys.2017.00867)
Supplement: Supplementary Figure I — A schematic showing the approximate region of the organum vasculosum of the laminae terminalis (OVLT) in which Fos-positive cell nuclei were counted. The photomicrograph is an example of a section counted. The approximate rostrocaudal level relative to bregma is given in mm. [file Image1.PDF]

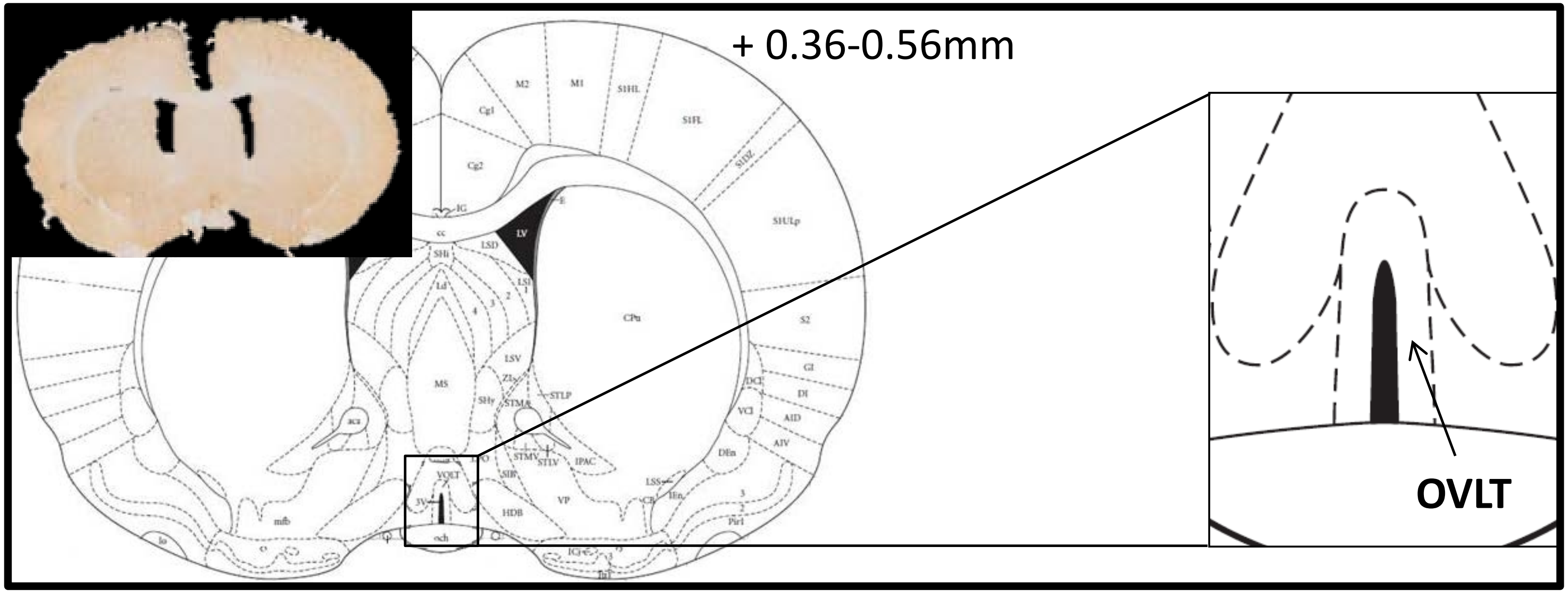

Supplementary figure I

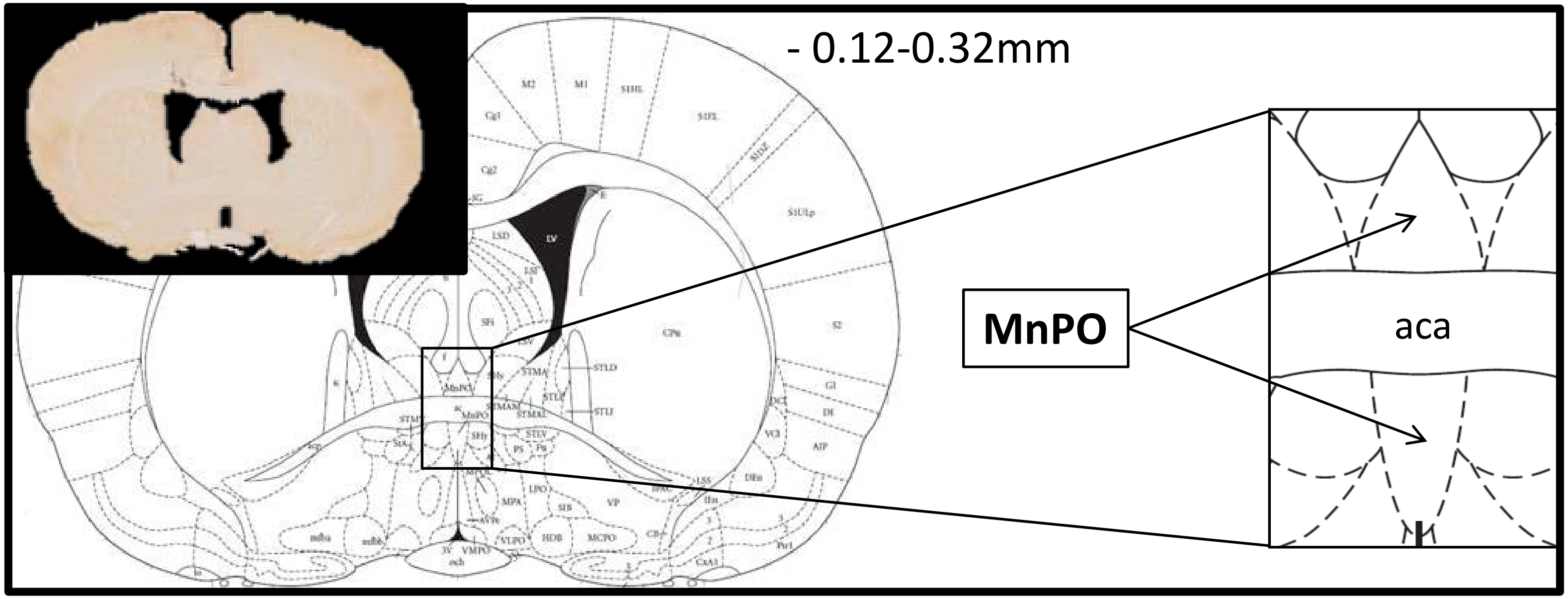

Supplementary figure II

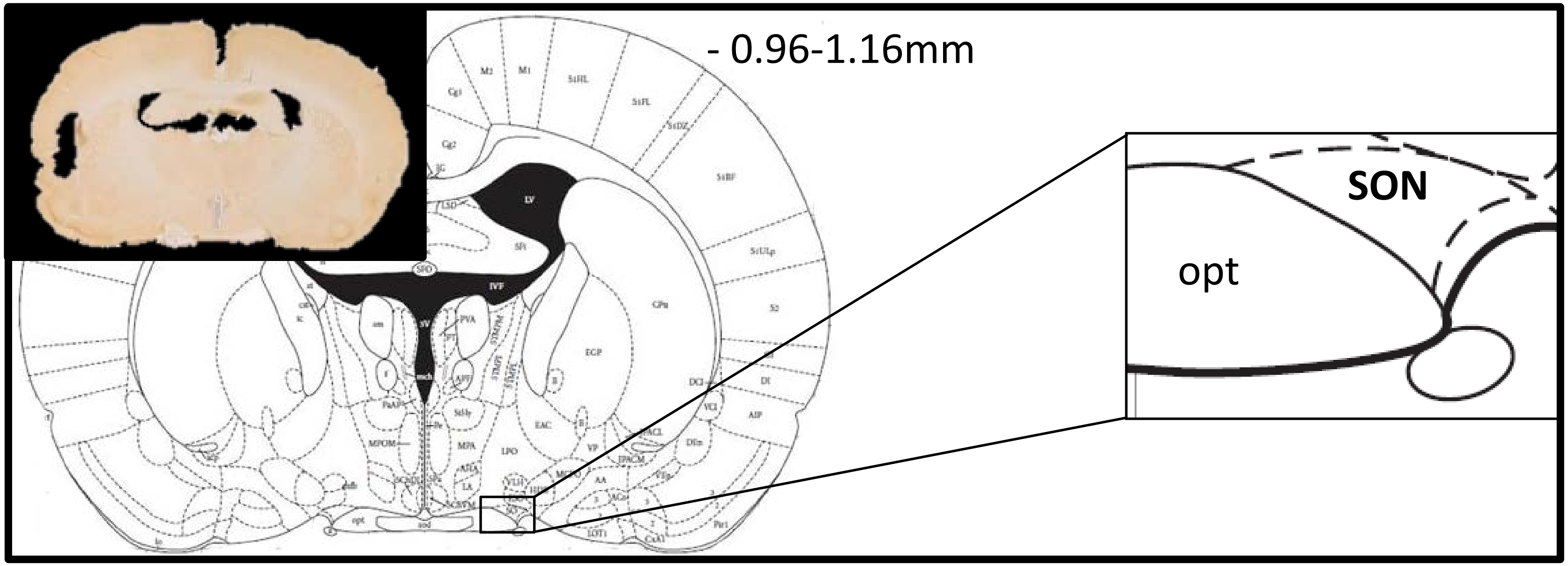

Supplementary figure III

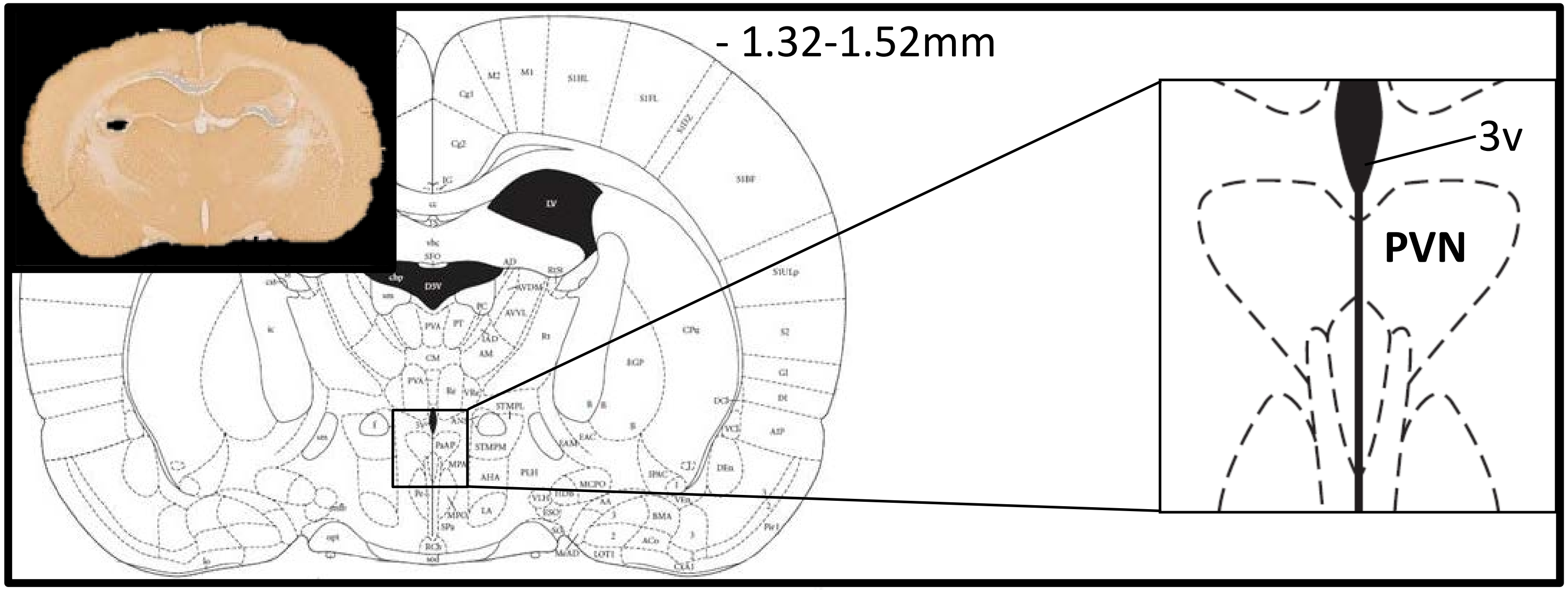

Supplementary figure IV

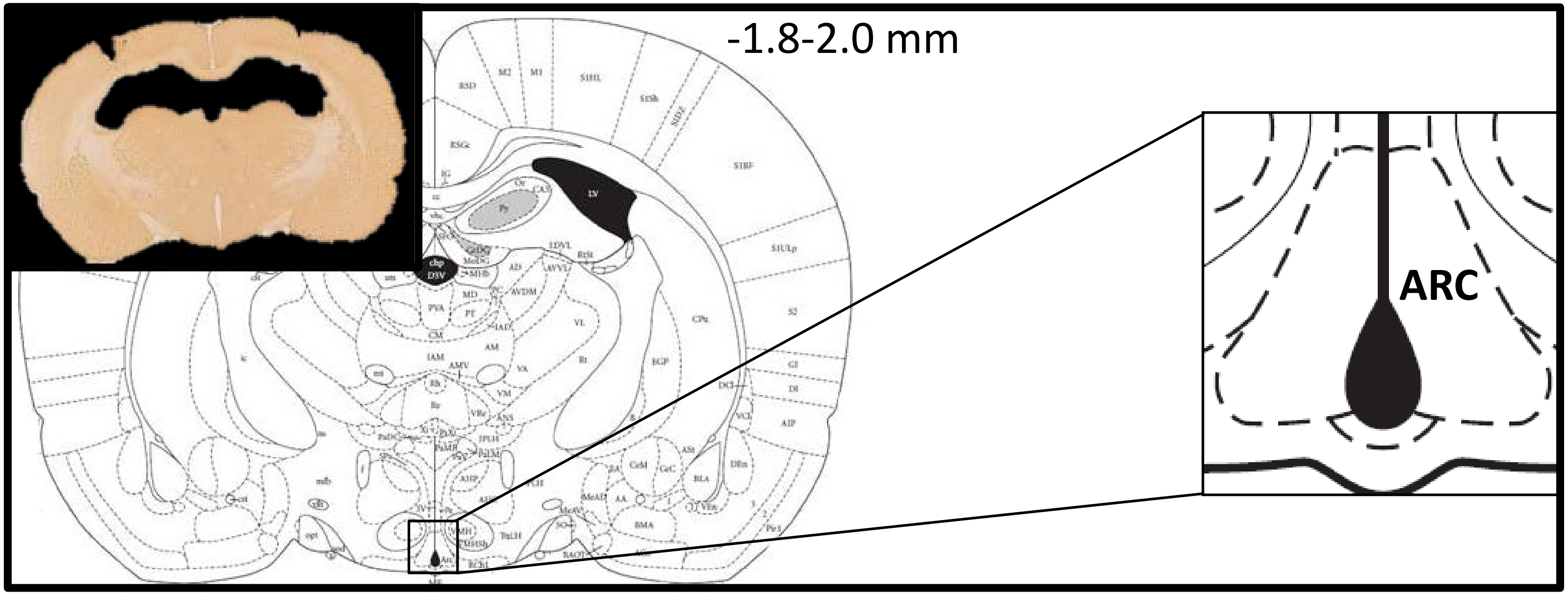

Supplementary figure V

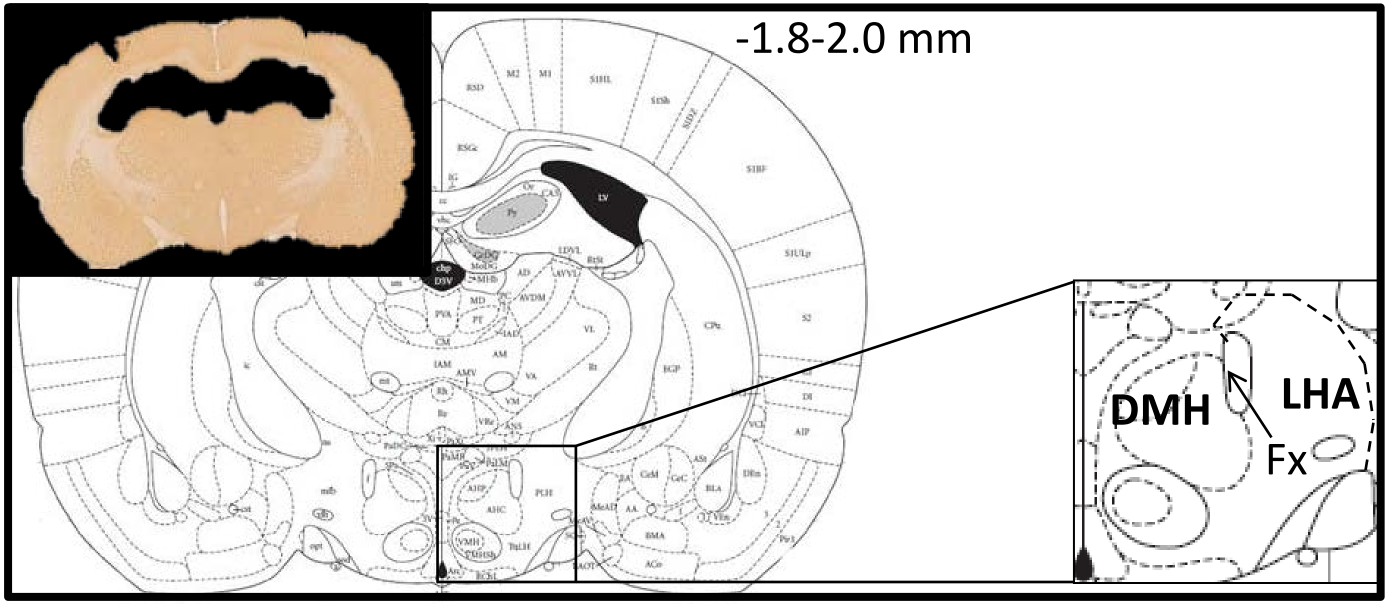

Supplementary figure VI

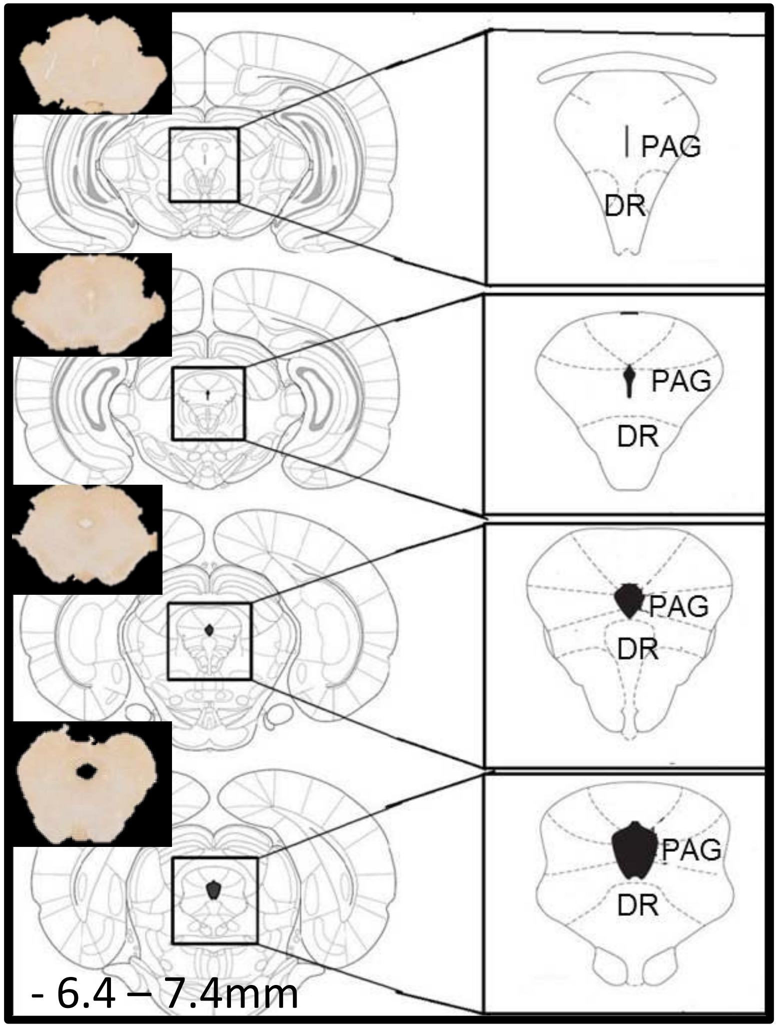

Supplementary figure VII

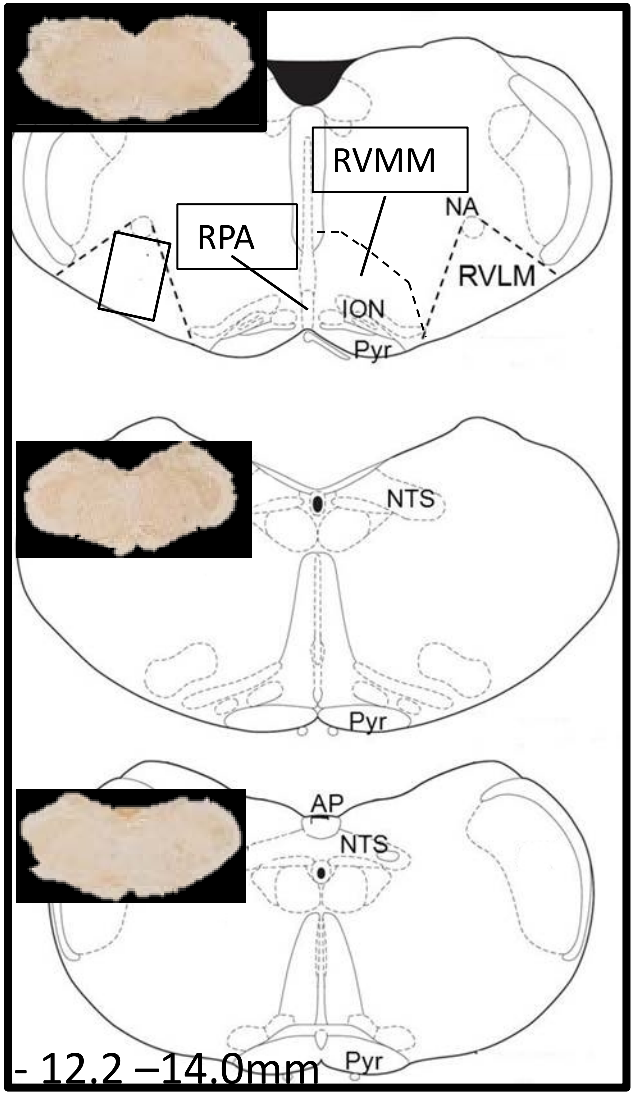

Supplementary figure VIII
