# Supplementary figures and images for: High Fat Diet Decreases Neuronal Activation in the Brain Induced by Resistin and Leptin
Source: Front Physiol. 2017 Nov 28;8:867. doi: 10.3389/fphys.2017.00867 (PMC5712409; doi:10.3389/fphys.2017.00867)

**ND**

**HFD**

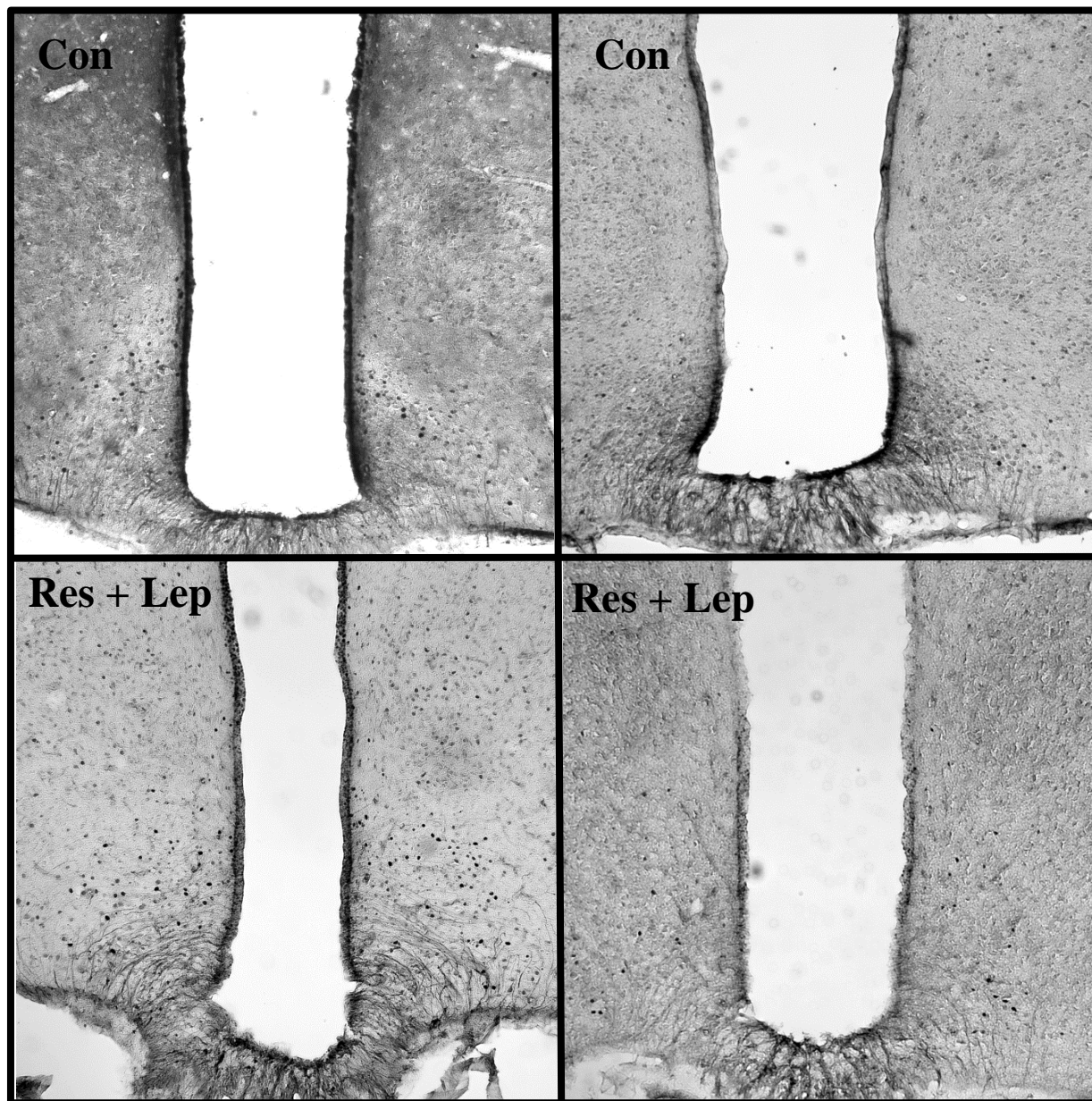

Supplement: Supplementary file 2 [file Presentation1.PDF]
